# Trends in paediatric antiseizure-medication use and costs in France, 2014–2023: a nationwide population-based analysis

**DOI:** 10.1016/j.lanepe.2026.101594

**Published:** 2026-02-03

**Authors:** Rima Nabbout, Annaëlle Santilli, Louise Tyvaert, Cyril Schweitzer, Louis Maillard, Mathieu Kuchenbuch

**Affiliations:** a Reference Centre for Rare Epilepies, Department of Paediatric Neurology, Necker Enfants Malades Hospital, APHP, Member of European Reference Network EpiCARE, Universite Paris Cité, 24 Boulevard du Montparnasse, 75015, Paris, France; b Institut Imagine, INSERM UMR 1163, Translational Research on Neurological Diseases, Université Paris Cité, Paris, France; c Université de Lorraine, CHRU-Nancy, Service de Pédiatrie, Reference Centre for Rare Epilepsies, Membrer of European Reference Network EpiCARE, F-54000, Nancy, France; d Université de Lorraine, IMoPA, UMR CNRS 7365, F-54000, Nancy, France; e Université de Lorraine, CHRU-Nancy, Service de Neurologie, Reference Centre for Rare Epilepsies, Membrer of European Reference Network EpiCARE, F-54000, Nancy, France

**Keywords:** Paediatric epilepsy, Antiseizure medications, Drug utilization trends, Generic substitution, Valproate safety, Pharmacoepidemiology

## Abstract

**Background:**

Epilepsy is the commonest chronic neurological disorder of childhood. In France, pregnancy-prevention rules and generic policies aim to improve safety and affordability of anti-seizure medications (ASM), but their paediatric impact is unknown. Tracking these patterns is key to assess policy effectiveness and to ensure safe, equitable, and sustainable access to treatment. We aimed to characterise nationwide temporal trends in paediatric ASM use, costs, generic uptake, and sex differences in France from 2014 to 2023.

**Methods:**

We conducted a retrospective drug-utilisation study using open-access OpenMedic files from the French National Health Data System. All dispensing of 24 ASMs to individuals <18 years during 2014–23 were retrieved by product code. Annual users, boxes, and costs were summarised; temporal trends used Spearman tests; multivariable logistic regression modelled sex differences; LASSO selected determinants of generic dispensing.

**Findings:**

Over the decade, 2,015,504 children consumed 15,748,141 ASM boxes, costing €274.4 million. Annual users increased 24% (from 174,889 to 216,607). Third-generation agents rose 70% (from 98,659 users per year to 168,290), whereas first- and second-generation agents fell 48% (from 1579 to 823) and 28% (from 87,929 to 63,038). Valproate use fell 37% overall (from 58,845 to 37,014) and 62% in girls (from 23,480 to 8975); lamotrigine and levetiracetam rose 49% (from 29,404 to 43,847) and 77% (from 37,290 to 65,940), respectively. Generics accounted for 11.2% of dispensing in 2014 (191,551 boxes) vs 20.2% in 2023 (455,934 boxes). Prescriptions by private psychiatrists and use of gabapentin, pregabalin, lamotrigine, and levetiracetam independently predicted generic uptake. ASM with available generic substitution would have cut 2023 spending by 8% (€3.88 million).

**Interpretation:**

Paediatric ASM practice in France is rapidly aligning with safer, newer ASM and sex-specific risk mitigation, yet generic penetration lacks. Targeted substitution strategies could release funds for innovative therapies without compromising seizure control.

**Funding:**

None.

**Research in context**

**Evidence before this study**

We searched PubMed and Embase (Jan 1 2010–Feb 15 2025) for population-based paediatric studies on antiseizure-medication utilisation and policy interventions (“antiepileptic” OR “antiseizure” AND “children” AND “regulation” OR “policy”). We found six national investigations (UK, France, Norway, South Korea, USA, Australia). Four reported sex-specific trends; none quantified generic uptake.

**Added value of this study**

Using 10 years of exhaustive reimbursement data (>2 million children), we quantify how pregnancy-prevention measures and generic incentives reshaped paediatric prescribing, reduced valproate exposure in girls, and revealed an 8% avoidable spending margin through generics (€3.88 million).

**Implications of all the available evidence**

Regulatory packages coupling teratogenic-risk mitigation with active promotion of generics may contribute to improving both safety and affordability. Real-world reimbursement databases are an effective, scalable tool to track progress towards WHO Essential Medicines and SDG 3.8 targets, and to flag persisting socioeconomic and sex inequities that warrant corrective action.

## Introduction

Epilepsy is among the most frequent chronic neurological diseases, affecting over 50 million people worldwide, with a major burden in low- and middle-income countries.^1^ It is diagnosed after recurrent unprovoked seizures or when a syndrome is identified,^2^ and strongly impacts quality of life. Anti-seizure medications (ASMs) remain the cornerstone of epilepsy management. They aim to stop seizures targeting underlying specific mechanisms or aetiologies. When appropriately chosen, i.e., to maximise seizure control while minimising adverse effects, antiseizure medications can achieve seizure freedom in up to 70% of patients.^3^ Their development has proceeded in successive waves since the early 20th century, leading to ∼25 marketed products in France (Supplementary Figure S1, Supplementary Table S2).

ASM choice takes into account a series of criteria including the type of seizures, epilepsy syndrome, age, sex, aetiology, comorbidities. This choice was highly impacted during the last decade by new ASM developments and reported teratogenicity risk. Concerns about malformation and neurodevelopmental risks have prompted strict risk-minimisation measures: sodium valproate has been progressively restricted since 2014–17^4^ and topiramate since 2022,^5^ limiting their use in women of child-bearing age to refractory epilepsy only.

We analysed nationwide dispensing records in the open-access OpenMedic repository of the French National Health Data System between 2014 and 2023 in order to characterise temporal trends in paediatric ASM prescribing in France, with a focus on (1) the impact of teratogenicity-related safety actions and (2) shifts towards generic formulations.

## Methods

### Study design and population

We used OpenMedic, the open-access extract of the French National Health Data System, which covers ≈99% of the population and provides anonymised dispensing data from community pharmacies. Files include product code, brand, pack size, patient sex and age-band, and prescriber specialty. Further details appear in Supplementary S1.

### Procedures

All dispensing of ASMs to individuals <19 years between 1 January 2014 and 31 December 2023 were retrieved. We first compiled an inventory of every branded and generic ASM marketed during the window (Supplementary S2), annotated for dose, pharmaceutical form, pack size and French CIP13 code, a product-level identifier distinct from the International Non-Proprietary Name (INN), which denotes the active substance. Presentations sharing an CIP13 were collapsed at molecule level and classified as first-, second- or third-generation; third-generation drugs were subdivided into early (1990–1999), mid (2000–2009) and late (2010–2025) subclasses. For each calendar year we extracted: (i) the number of children exposed to each ASM, (ii) the total number of packs dispensed, and (iii) the corresponding reimbursed expenditure.

### Statistical analysis

Continuous variables are reported as median (IQR), categorical variables as counts (%). Temporal trends were assessed with Spearman rank tests and ordinary least-squares regression. Annual counts of users and packs were over-dispersed; therefore, negative-binomial regression was applied, yielding yearly rate ratios (RRs) with 95% Cis (Supplementary S3). Sex differences in molecule choice were examined with multivariable logistic regression adjusting for calendar year and prescriber speciality. Least-absolute-shrinkage-and-selection operator regression (LASSO), used to counter multicollinearity, identified factors independently associated with generic dispensing. Potential savings from wider substitution were gauged by recalculating 2023 expenditure under a counter-factual scenario in which every originator pack was replaced by the median-priced generic of identical substance, dose, form and pack size; the difference from observed spend represents avoidable cost. Because cannabidiol's launch produced a single-year price spike, we initially fitted a log-linear Poisson model excluding this agent, but due to over-dispersion and the continuous nature of expenditure, we retained a Gamma regression with a log link as the more appropriate final model. Further sensitivity checks comprised leave-one-year-out refits of logistic models and calculation of E-values to estimate the strength of unmeasured confounding required to nullify observed associations.

All analyses were performed with R (version 4.3); two-sided p values < 0.05 were considered statistically significant.

### Ethics approval

The study uses anonymised, open-access administrative data (OpenMedic); therefore, ethics committee approval and informed consent are not required under French law.

### Role of the funding source

This study was not supported by any funding source.

## Results

### Participants

Between 2014 and 2023, we identified a total of 2,015,504 ASM users (an individual could be identified as a user in multiple calendar years), yielding a cumulative 15,748,141 treatment packs dispensed in the period (Supplementary Figure S2). On a yearly basis, this corresponded to a median of 202,706 [193,010–205,272] individuals and 1,583,942 [1,547,329–1,603,791] boxes dispensed. The median annual sex ratio among paediatric ASM users was 1 [0.97–1.002]. The medications delivered covered 24 different ASM. The most frequently used galenic forms were tablets (n = 1,099,881; 54.6%), granules (n = 364,340; 18.1%), oral solutions (n = 326,518; 16.2%), and capsules (n = 224,765; 11.2%). First-generation ASMs accounted for 0.6% of consumers (n = 11,242), while second- and third-generation ASMs represented 34.8% (n = 7,01,368) and 64.6% (n = 1,302,894), respectively. Among those treated with third-generation ASMs, 60% (n = 781,465) received drugs licensed in the 1990s, 38% (n = 495,455) in the 2000s, and only 2% (n = 25,974) in the 2010s (Supplementary Figure S3). The five most prescribed ASMs were valproic acid (n = 548,079; 27.2%), lamotrigine (n = 527,684; 26.2%), levetiracetam (n = 334,000; 16.6%), carbamazepine (n = 112,734; 5.6%), and topiramate (n = 110,680; 5.5%, Supplementary Figure S4). Valproic acid and carbamazepine together accounted for 94% of second-generation ASM use (78% and 16%, respectively), while lamotrigine, levetiracetam, and topiramate represented 75% of third-generation prescriptions (41%, 26%, and 9%, respectively).^6^ Hospital physicians issued 1,177,432 dispensing (58.4%), whereas the ambulatory sector accounted for the remaining 41.6%: general practitioners 467,358 (23.2%), neurologists 105,250 (5.2%), paediatricians 51,622 (2.6%), and psychiatrists 21,123 (1.1%). Prescriber specialty was unrecorded for 191,940 dispensing (9.5%; Supplementary Figure S5).

### Evolution of ASM between 2014 and 2023

Between 2014 and 2023, the number of annual paediatric ASM users increased from 174,889 to 216,607 (+24%; ρ = 0.86; p = 0.0056), while the French population aged <20 years slightly declined, corresponding to a ≈25–28% relative rise in treated prevalence (Supplementary Table S3). Negative-binomial regression confirmed a significant rise in paediatric ASM users of 1.7% per year (rate ratio 1.017, 95% CI 1.010–1.025) vs 0.7% per year for boxes (1.007, 1.004–1.010), supporting expansion driven by newly treated patients rather than higher per-patient volumes.

**Fig. 1:**
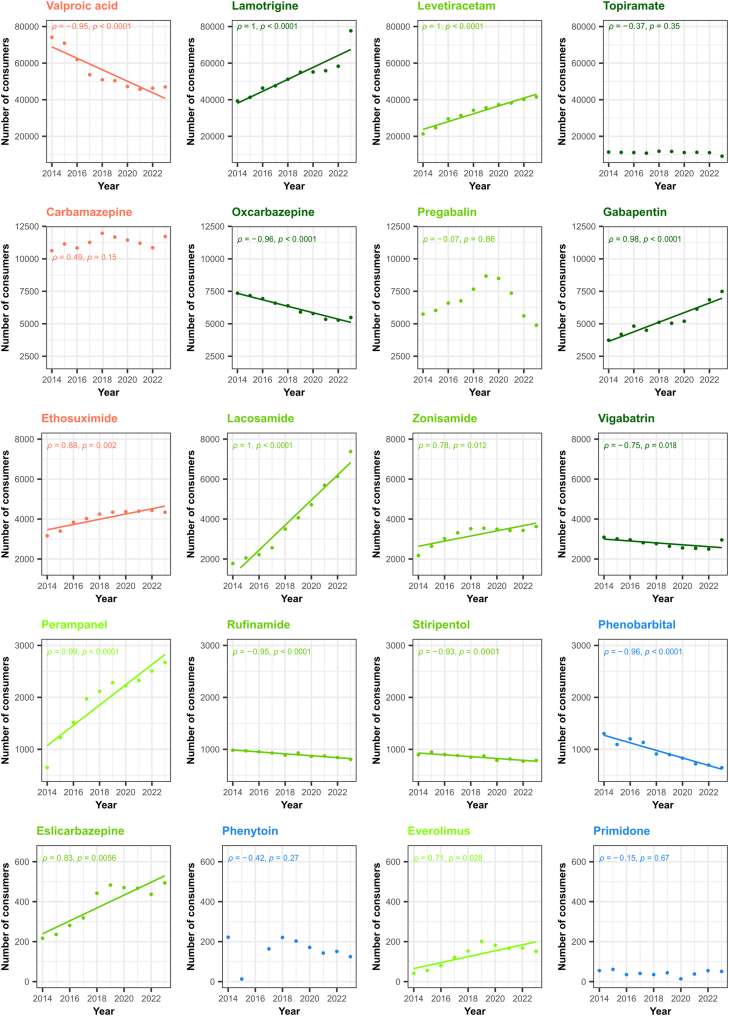
**Trends in annual paediatric anti-seizure medication (ASM) consumption in France, 2014–2023, by pharmacological generation.** Number of children exposed to each ASM by calendar year. Colours indicate pharmacological generation: blue = first generation; orange = second generation; light, medium, and dark green = third generation (licensed in the 1990s, 2000s, and since 2010, respectively). Y-axis scales are consistent within each row. Within each row, the y-axis scale is harmonised to allow comparison across molecules. Declines are observed for valproic acid, carbamazepine, oxcarbazepine, phenobarbital, and rufinamide, whereas lamotrigine, levetiracetam, lacosamide, and perampanel show sustained increases.

First-generation use fell from 1579 to 823 and second-generation use from 87,929 to 63,038, whereas third-generation prescriptions increased from 98,659 to 168,290, with parallel rises for ASM launched in the 1990s (from 64,870 to 102,641), the 2000s (from 33,099 to 59,512), and the 2010s (690–6137). These trends were all strongly monotonic (ρ ≥ 0.9). At the molecule level, listed below in descending order of overall prescription volume, annual users decreased by 37% for valproic acid, 26% for oxcarbazepine, 4.3% for vigabatrin, 18% for rufinamide, 12% for stiripentol, and 50% for phenobarbital. Conversely, lamotrigine prescriptions rose by 49%, levetiracetam by 77%, and newer add-ons (lacosamide, perampanel, eslicarbazepine, and everolimus) each increased by more than 125% (Fig. 1).

Over the same period, prescriptions issued by hospital practitioners increased from 102,079 (54.3% of the consumers) to 145,932 (62.9%), a relative increase of 43% (Supplementary Figure S6). Prescriptions issued by private psychiatrists increased more than four-fold (from 1180 (0.6%) to 6242 (2.7%), +429%). In contrast, those by general practitioners felt from 55,201 (29.3%) to 39,559 (17.0%) (−28.7%). Counts for office office-based paediatricians (∼2.5%) and neurologists (∼5.2%) remained stable throughout the study period (Supplementary Figure S6).

### Prescription of ASM according to sex

**Fig. 2:**
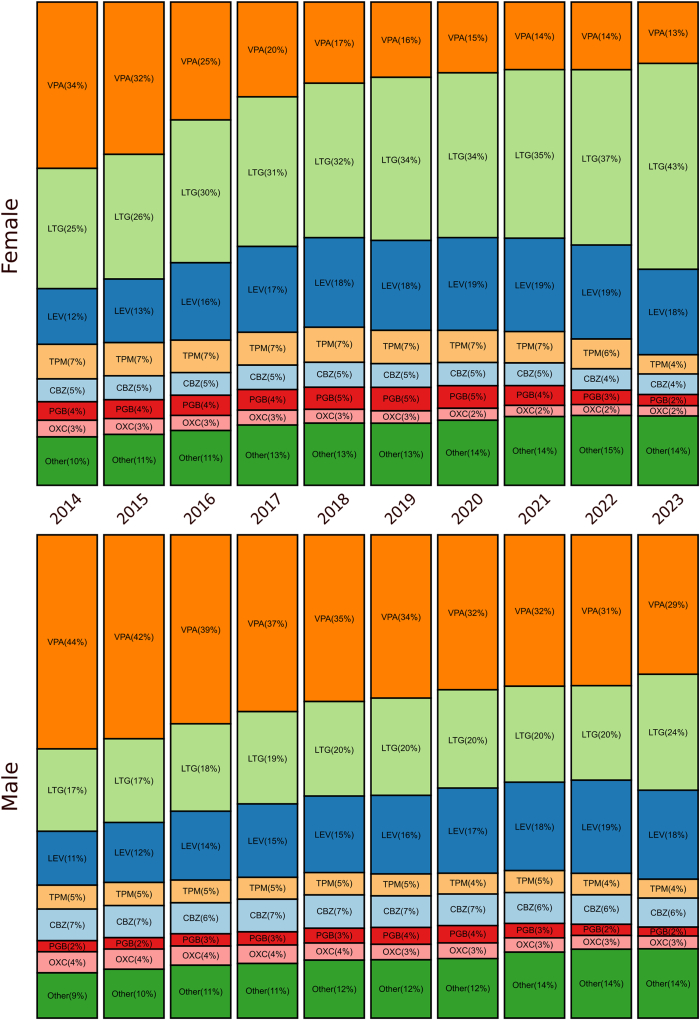
**Sex-specific evolution of anti-seizure medication (ASM) use in France, 2014–2023.** Stacked proportions of annual prescriptions by sex. In girls, valproate (VPA) declined from 34% to 13%, while lamotrigine (LTG) and levetiracetam (LEV) increased from 25% to 43% and from 12% to 18%, respectively. Boys showed the same overall direction of trends, decreasing valproate and increasing lamotrigine and levetiracetam use, but with smaller amplitude of change and a nearly identical ranking of molecules over time. Boys showed a similar but less pronounced pattern. Abbreviations: BRV = brivaracetam; CBZ = carbamazepine; CBD = cannabidiol; CNB = cenobamate; ESL = eslicarbazepine; ESM = ethosuximide; EVER = everolimus; FFA = fenfluramine; GBP = gabapentin; LCM = lacosamide; LEV = levetiracetam; LTG = lamotrigine; OXC = oxcarbazepine; PB = phenobarbital; PER = perampanel; PGB = pregabalin; PHT = phenytoin; PRM = primidone; RFM = rufinamide; STP = stiripentol; TPM = topiramate; VGB = vigabatrin; VPA = valproic acid; ZNS = zonisamide.

**Fig. 3:**
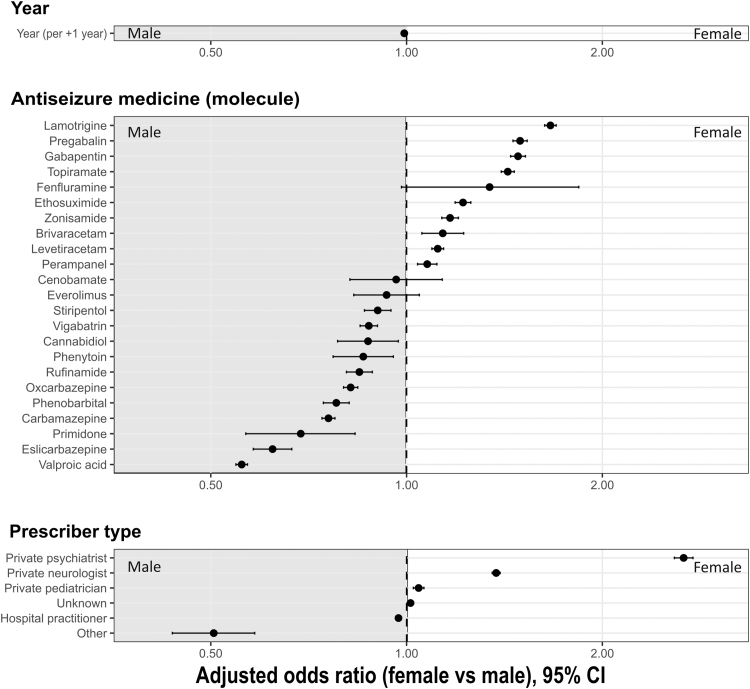
**Sex differences in anti-seizure medication (ASM) prescriptions.** Forest plot showing odds ratios (ORs) with 95% confidence intervals (CIs) for the probability of being female rather than male among consumers of anti-seizure medications (ASMs), according to the prescribed ASM, calendar year, and prescriber type. Estimates were obtained from a logistic regression model weighted by the number of consumers, adjusted for year (continuous), ASM, and prescriber specialty. An OR greater than 1 indicates a higher relative proportion of females, whereas an OR less than 1 indicates a higher relative proportion of males.

The proportion of girls increased steadily over the decade, driving the male-to-female ratio down from 1.03 in 2014 to 0.91 in 2023 (ρ = −0.94, p < 0.0001). In absolute terms, the number of females rose from 92,394 (49.1%) to 121,181 (52.2%). Among girls, the annual number of valproate prescriptions dropped from 34% to 13% between 2014 and 2023. Conversely, those of levetiracetam and lamotrigine increased from 12% to 18% and from 25% to 43%, respectively (Fig. 2). Among boys, a similar trend is observed with a decrease in valproate from 44% to 29%, and an increase in lamotrigine from 17% to 24% and levetiracetam from 11% to 18%. Leave-one-year-out refits showed that valproate remained 55–59% less likely to be prescribed to females than to males, odds ratios for ‘female sex vs male sex, valproate vs all other ASMs’ ranged from 0.417 to 0.449, and the only discernible uptick appeared when 2017 (the first year of reinforced restrictions by the French agency for the prescriptions in girls) was omitted (from 0.433 to 0.442; Supplementary Figure S7). E-values of ≥2.3 show that even this small gap would require an unmeasured confounder to more than double the odds of both valproate exposure and being female to overturn the association. In multivariable analyses, prescriptions for valproate, eslicarbazepine, primidone, carbamazepine, phenobarbital, oxcarbazepine, rufinamide, phenytoin, cannabidiol, vigabatrin, and stiripentol were significantly more frequent in males (ORs 0.56–0.90). By contrast, lamotrigine, pregabalin, gabapentin, topiramate, ethosuximide, zonisamide, brivaracetam, levetiracetam, and perampanel were preferentially prescribed to females (ORs 1.08–1 .66). Females also received more prescriptions from private psychiatrists, neurologists, and paediatricians, whereas males were more often treated in hospitals or by other providers. Increasing calendar year was independently associated with a small shift toward male prescribing (OR 0.992, 0.991–0.993 per year) (Fig. 3, Supplementary Table S4).

### Prescription of ASM according to generic formulation

**Fig. 4:**
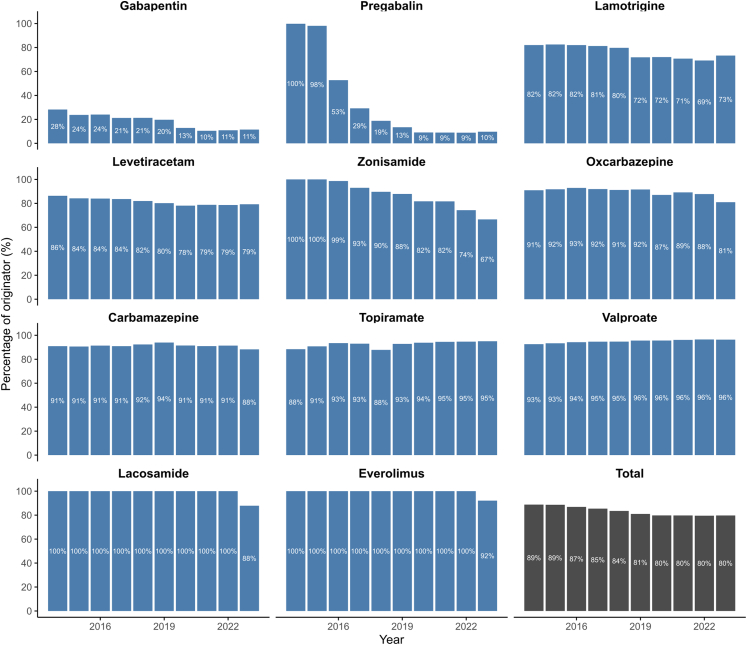
**Uptake of generic vs originator anti-seizure medications (ASMs) in France, 2014–2023.** Percentage of prescriptions accounted for by the originator brand (y-axis) over time (x-axis), stratified by molecule. Generic uptake was highest for gabapentin (GBP) and pregabalin (PGB), while persistently high brand use was mainly observed for zonisamide (ZNS). “Total” indicates the overall originator share across all ASMs with available generics. Abbreviations: VPA = valproate; LTG = lamotrigine; LEV = levetiracetam; CBZ = carbamazepine; OXC = oxcarbazepine; GBP = gabapentin; PGB = pregabalin; LCM = lacosamide; PER = perampanel; RFM = rufinamide; STP = stiripentol; PB = phenobarbital; PRM = primidone; VGB = vigabatrin; ESM = ethosuximide; CNB = cenobamate; BRV = brivaracetam; EVER = everolimus; ESL = eslicarbazepine; ZNS = zonisamide.

**Fig. 5:**
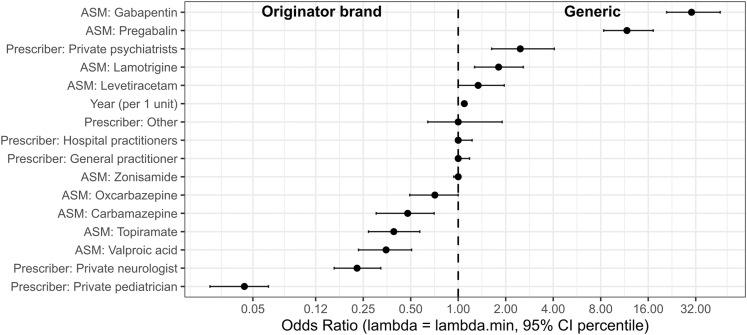
**Factors associated with generic uptake of antiseizure medications.** Forest plot of odds ratios (ORs) with 95% bootstrap confidence intervals for the association between individual anti-seizure medications (ASMs), type of prescriber, and calendar year with the probability of receiving a generic prescription. Estimates were obtained from a LASSO logistic regression model with 500 bootstrap replications. Zonisamide was used as the reference ASM, and general practitioners as the reference prescriber. ORs greater than 1 indicate higher odds of generic prescription (generic side), whereas ORs less than 1 indicate lower odds (originator side). CI = confidence interval; ASM = anti-seizure medication. These odds ratios primarily capture cross-sectional differences in generic uptake between molecules rather than large within-molecule changes over time.

Among the 24 ASMs, 12 (50%) had at least one generic version available on the French market. These 12 ASM were marketed in 62 distinct presentations, each a unique pairing of dosage strength (tablet strength or syrup concentration) and pack size (tablet count or bottle volume), 18 of these presentations (29%) lacked a generic equivalent. Generic versions represented 8.3% of all ASM dispensing, i.e., 1,308,168 boxes in total. ASM that had a generic counterpart offered a far wider range of box types (29.5 [13–42]) than brand-only ASM (4 [1–5.5], p < 0.0001), by contrast, the number of galenic presentations was similar (1.5 [1–2] vs 2 [2–3], p = 0.082). Each year a median of 38,838 [26,162–41,252] individuals received a generic ASM, accounting for 16.9% of all ASM users. This share rose steadily from 11.2% in 2014 to 20.2% in 2023 (ρ = 0.96 [0.82–0.99], p < 0.0001), alongside an increase in the number of agents available as generics (from eight to 11; Fig. 4). In LASSO models, generic uptake was higher for gabapentin and pregabalin, and to a lesser extent for lamotrigine and levetiracetam; it increased over calendar time. Lower uptake was observed for valproate and in prescriptions issued by private neurologists and paediatricians compared with GPs (Fig. 5; Supplementary Table S4).

### Cost analysis and savings potential through generics

Between 2014 and 2023, cumulative paediatric expenditure on ASMs in France totalled €274,376,756, with a median annual expenditure of €25,508,934 [25,257,185–25,817,819] per year (Supplementary Figure S8). Most of the decade spending was flat (−0.9 [−1.6 to 1.1] % annually). However, the landscape shifted abruptly in 2023, when expenditure jumped 80.7%, from €25,235,697 to €45,599,396. Cost nonetheless escalated at the individual-medicine level. In 2023, the median annual cost per patient and per ASM was €136 [IQR 51.5–455.1] and increased stepwise with newer ASM generations: from €22.0 [7–56] for first-generation, €37.1 [13–80] for second-generation, and €303.3 [89.1–783.1] for the third-generation (Supplementary Figure S9). Within the third generation, costs increased with the decade of market entry: €72.0 [IQR 29–148] for ASM launched in the 1990s, €214.9 [IQR 115–442] in the 2000s, and €1545 [IQR 238–6604] for ASM introduced since 2010. Trend tests confirmed a significant monotonic increase (Jonckheere-Terpstra p < 0.0001). As a result, ASMs launched in the 2010s already accounted for 52.9% of total ASM spending in 2023 while representing only 2.6% of prescriptions. Of the additional €20,363,699 in 2023, €19,158,200 (94.1%) were attributable to the market entry of cannabidiol and €874,742 (4.3%) to that of fenfluramine. When cannabidiol was excluded, a Gamma regression with a log link showed no significant temporal trend in total paediatric ASM expenditure over the study period (rate ratio ≈ 1.01 per year, 95% CI 1.00–1.01; p = 0.11), confirming the overall stability of spending between 2014 and 2023 (Supplementary Table S5). Continuous generic substitution also curbed expenditure, saving a cumulative €3,239,433, i.e., €283,285 [261,787–405,174] per year and rising almost linearly from €204,989 in 2014 to € 457,491 in 2023 (ρ = 0.96, p < 0.0001). Under a counterfactual all-generic scenario, 2023 ASM expenditure would have fallen by €3,875,968, representing an 8.5% reduction, highlighting the untapped savings still available.

## Discussion

This nationwide cohort offers the first population-based picture and trends over the last decade of paediatric ASM use in France. By linking community and hospital dispensing for almost every child living in the country, we captured both long-term trends and abrupt policy shocks. Three signals emerge: a decisive move from first- and second-generation drugs to compounds licensed in the 1990s; increasing sex-specific changes driven by teratogenicity warnings; and a still-cautious adoption of generic formulations despite broad market availability. These conclusions are unlikely to be artefactual: negative-binomial and Poisson sensitivity models reproduced the descriptive trends, while our leave-one-year-out and E-value analyses showed that neither single-year anomalies nor plausible unmeasured confounding could overturn the sex-specific decline in valproate use. Our findings confirm that third generation now dominate French practice: lamotrigine, levetiracetam, oxcarbazepine, and topiramate together account for more than two-thirds of all boxes dispensed in 2023. Similar transitions have been reported in Japan, Norway, and the UK, where newer drugs are valued for lower enzyme-induction, simpler titration, and improved tolerability.^7^ Cross-national comparisons, however, reveal striking differences in rank order. Lamotrigine is first-line in France and Scandinavia, whereas levetiracetam leads in the Netherlands, China, and much of East Asia, largely because HLA-B∗1502-related cutaneous toxicity limits lamotrigine uses in those regions.^8^^,^^9^ Such discrepancies underscore the continuing influence of local pharmacogenomic profiles, reimbursement rules, and medical-education.

Pregnancy-prevention measures (2014–17) were followed by a 37% drop in paediatric valproate use, greater in girls than in boys, consistent with UK and Scandinavian data.^10^^,^^11^ More recently, French authorities also extended restrictions to men of reproductive age. These regulatory changes, which include initiation and annual renewal patient-specialist agreement forms may also have contributed to the redistribution of prescribers observed over the study period. Although these requirements apply only to a small subset of the paediatric population, they may have led to a gradual decline in ASM prescribing by general practitioners and a corresponding increase among hospital-based prescribers, together with other contextual factors such as the expanding range of available ASMs and a growing trend toward more individualised management of complex cases in specialised care. Topiramate presents a different picture. Dispensed to fewer than 20,000 paediatric patients annually in France, it has shown only a modest decrease after the ANSM's 2022 warning about foetal teratogenicity and neurodevelopmental risk.^5^ The limited effect is unsurprising, because in France, topiramate is reserved mostly for pharmacoresistant epilepsies, as Lennox-Gastaut or Dravet syndromes, in which pregnancy planning is an exceptional, rather than routine, issue. Still, data illustrate how recency of guidance and clinical context shape uptake of risk-minimisation measures. Lamotrigine and levetiracetam have filled much of the therapeutic gap left by valproate. In girls, lamotrigine prescriptions rose by 18% and levetiracetam by 4% over last decade. Both drugs have favourable reproductive–safety profiles, although lamotrigine has been associated with a small increase in isolated orofacial clefts and levetiracetam with conflicting neurodevelopmental signals.^12^^,^^13^ These changes in prescribing practices may be also based on additional data on its superiority to levetiracetam and zonisamide for time to 12-month remission in focal epilepsy in adults, whereas valproate remained superior to levetiracetam for generalised epilepsies.^14^ The dilemma for female patients with generalised epilepsies, weighting valproate efficacy vs teratogenicity therefore persists, and emphasises the need for novel, pregnancy-safe broad-spectrum ASMs.^15^ Over the decade, the proportion of treated girls increased steadily, driving the male-to-female ratio down from 1.03 to 0.91. This evolution does not appear to reflect a demographic change in the French paediatric population, whose sex distribution has remained stable over the past decade.^16^ Rather, it may result from subtle differences in diagnosis, treatment initiation, or follow-up between boys and girls. However, this hypothesis would require individual-level clinical data that are not available in our dataset. This population-level shift is distinct from the adjusted year-on-year trend described in our multivariable model, which reflects a very small residual tendency toward male prescribing once prescriber and drug effects are accounted for.

Generics covered only 20% of paediatric users in 2023, vs >90% for other drug classes. Concerns about therapeutic index and seizure risk still deter clinicians,^17^ although trials such as EQUIGEN have shown equivalence when substitution is supervised.^18^ The American Epilepsy Society now states that initiation of therapy can be with either originator or generic, while conversion of a seizure-free child should be individualised.^19^ The remaining barriers are therefore largely logistical (multiple manufacturers, intermittent supplies) and psychological (physicians, patients and pharmacists distrust of lower-priced products). Removing these obstacles, and guaranteeing continuity of a single formulation, could unlock substantial savings in high-income settings. In our study, differences in generic uptake likely reflect molecule-specific contexts rather than uniform prescriber behaviour. For instance, gabapentin and pregabalin, frequently prescribed off-label for paediatric neuropathic pain, naturally shows higher generic penetration than ASMs restricted to epilepsy use. Although our model pooled all ASMs to identify common determinants, future work could stratify molecules by their baseline proportion of generic use to explore whether predictors differ between high- and low-uptake drugs.

Annual ASM expenditure was remarkably flat from 2014 to 2022, oscillating fluctuating by only ≈ −0.9% per year. In 2023, however, spending jumped by 80%, driven almost largely by the launch of pharmaceutical-grade cannabidiol, which added €19.2 million in a single year. In US, Moura and colleagues analysed 2010–18 Medicare, Medicaid, and commercial claims and showed that mean annual spending of ASM per patient with epilepsy rose by 8.8%, driven chiefly by the rapid adoption of high-cost branded ASMs and rising unit prices.^15^ A complementary analysis by Sánchez Fernández et al. tracked 2006–21 US retail sales and found that raw ASM expenditure more than doubled, with newer agents (e.g., perampanel, lacosamide) accounting for the bulk of the increase.^20^ These trends illustrate what France may face if prices rise unchecked and underscore the value of early generic substitution. Continuous generic substitution has already shaved €3.2 million from the French bill over the decade and, in a counterfactual all-generic scenario, would have cut 2023 expenditure by a further 8.7%. Moreover, the large deficit of the health security system will be worsened by the upcoming arrival of gene and RNA-based therapies in epilepsy that are expected to significantly intensify financial pressures on the healthcare system. A leading candidate is zorevunersen, an antisense oligonucleotide (ASO) currently in late-stage development for Dravet syndrome. Extrapolating neuromuscular-drug prices suggests nationwide use of zorevunersen would add ≈ €130 million a year, while a one-off gene therapy for the ≈32 eligible 1-year-olds would demand a single upfront outlay of ≈€6 million (Supplementary Material). Such figures could prove only the beginning. Indeed, the overall diagnostic yield in genetic testing is estimated to 17%.^21^ If only a fraction reaches the market at current rare-disease prices and even if unit prices will decrease as gene-therapy technology matures, scales up, and moves into higher-prevalence indications, France's ASM budget could rise steeply within the coming decade. Ensuring robust generic uptake, through bio- and therapeutic equivalence between generics of a same molecule, strengthened substitution policies, and secure supply chains, together with transparent launch-price negotiations and outcome-linked payments, strategies already applied to ultra-expensive neuromuscular therapies,^22^ will be critical to preserving the budget needed for future innovative therapies in epilepsy. These mounting cost pressures in high-incomes countries foreshadow an even sharper inequity for low-income countries, where up to 90% of people with epilepsy already lack access to any antiseizure medication, generic availability averages <50% in the public sector, and the arrival of very high-priced gene and RNA-based therapies threatens to widen this treatment gap still further.^23^

OpenMedic's anonymised claims illustrate how France's open-data policy enables timely pharmaco-epidemiology and equity audits. Such files have revealed harmful drug–drug interactions years before formal reviews and allowed near-real-time monitoring of reimbursement equity.^24^, ^25^, ^26^ Once privacy safeguards are ensured, health-system data ethically belong to patients, and open access improves reproducibility and helps uncover coding errors.^27^^,^^28^ This study exemplifies how freely downloadable claims can yield policy-relevant insights while preserving confidentiality, and why continued expansion of France's open-data ecosystem will be essential for future therapy evaluation.

This study has some limitations. First, exposure was measured by the number of boxes dispensed rather than by counting unique patients; concomitant therapy and repeat dispensing may therefore inflate exposure, although the effect is probably small. Second, the database does not give precise indication on age, therefore we cannot stratify by age groups as we would have done for the 12–18 years group for Valproate for instance. In addition, there are no information on diagnosis (epilepsy syndrome) or medical history (type of seizures, age, sex, aetiology, comorbidities) allowing to confirm the indication and the choice of the therapy, which limits analyses with finer clinical granularity. We assumed epilepsy as the default, while recognising that some agents are frequently used in other indications, as gabapentin and pregabalin for neuropathic pain, topiramate for migraine, and lamotrigine or valproate for mood disorders. Such off-label use probably explains the higher proportion of generics for gabapentin and pregabalin. However, some of these indications are not highly frequent in paediatric age as valproate for mood disorders and the prescriptions by psychiatrists were very low (1%).

### Conclusion

Over the past decade, France has moved decisively towards safer, third-generation ASM and away from valproate in girls, while cautiously expanding generic use. Yet the sharp cost shock triggered by newest ASMs, and the looming entry of high-priced gene and RNA-based therapies, signal that today's health system equilibrium is fragile. If generic substitution is not accelerated and launch-price negotiations are not more tightly coupled to real-world value, the paediatric epilepsy budget could soon become unsustainable, widening treatment gaps both domestically and in resource-limited settings where 90% of people with epilepsy still receive no ASM at all.^29^ Harnessing France's open, anonymised claims infrastructure to monitor prescribing, prices, and outcomes in near real time offers a pragmatic path to safeguarding access: it enables rapid detection of inequities, rigorous evaluation of policy levers, and evidence-based re-investment of savings into genuinely transformative therapies. Strengthening education of physicians, families, and pharmacists on the safety and equivalence of generics will also be essential to overcome persistent reluctance. Coordinated action, linking risk-mitigation rules, robust generic supply chains, and outcome-linked payment models, will be critical to preserve the fiscal head-room required to deliver the next generation of innovative treatments to every child who needs them.

## Contributors

AS was involved in methodology, formal analysis, figure production, drafting the original manuscript, and reviewing and editing the manuscript. MK conceptualised the study and was involved in data extraction and curation, methodology, supervision, data analysis, verification and interpretation, visualisation guidance, project administration, and writing, reviewing, and editing the manuscript. RN conceptualised the study and contributed to supervision, data interpretation, and writing, reviewing, and editing the manuscript. LT, JJ, CS, and LM contributed to data interpretation and to reviewing and editing the manuscript. AS and MK had full access to and verified all underlying data. All authors approved the final version and were responsible for the decision to submit the manuscript.

## Data sharing statement

All raw dispensing data analysed in this study are publicly available from the Open Medic repository (https://www.data.gouv.fr/fr/datasets/open-medic-base-complete-sur-les-depenses-de-medicaments-interregimes/) and require no registration or permission. Because Open Medic does not contain individual-level identifiers, no additional patient-level data are available. The complete R scripts used for extraction, cleaning, analysis, and figure generation, together with the aggregated, de-identified analytic dataset (molecule-sex-age-year level), will be made available on reasonable request to the corresponding author after publication (m.kuchenbuch@chru-nancy.fr).

## Declaration of interests

RN reports personal fees for lectures from Biocodex, UCB Pharma SA, Jazz Pharmaceuticals, Eisai, Stoke, and Nutricia, outside the submitted work. LT reports personal fees for lectures and support for attending meetings or travel from Eisai, Bial, Angelini Pharma, Neuraxpharm, Jazz Pharmaceuticals, and UCB Pharma SA, outside the submitted work. LM reports personal fees for lectures from Angelini and Jazz Pharmaceuticals, support for attending meetings or travel from Angelini, and participation on a Data Safety Monitoring Board or Advisory Board for UCB Pharma SA, outside the submitted work. MK reports support for attending meetings or travel and receipt of hospitality-related benefits from Jazz Pharmaceuticals France and UCB Pharma SA, outside the submitted work. CS reports personal fees for lectures and support for attending meetings or travel from Sanofi, AstraZeneca, Novartis Pharma, PTC Therapeutics, and Parexel International, outside the submitted work. All other authors declare no competing interests.

## Appendix B Supplementary data

Abstract in French
