# Supplementary material for: Trends in paediatric antiseizure-medication use and costs in France, 2014–2023: a nationwide population-based analysis
Source: Lancet Reg Health Eur. 2026 Feb 3;63:101594. doi: 10.1016/j.lanepe.2026.101594 (PMC12887193; doi:10.1016/j.lanepe.2026.101594)
Supplement: Abstract in French [file mmc1.docx]

# Editor disclaimer

# This translation in French was submitted by the authors and we reproduce it as supplied. It has not been peer reviewed. Our editorial processes have only been applied to the original abstract in English, which should serve as reference for this manuscript.”

# Résumé

Contexte: L’épilepsie est la maladie neurologique chronique la plus fréquente chez l’enfant. En France, les règles de prescription vis-à-vis des risques tératogènes et les politiques relatives aux génériques visent à améliorer la sécurité et le coût des médicaments antiépileptiques (AED), mais leur impact en pédiatrie est inconnu. Le suivi de ces tendances est essentiel pour évaluer l’efficacité des politiques et garantir un accès sûr, équitable et durable aux traitements. Nous avons analysé les tendances nationales de prescription, de dépenses et de disparités entre les sexes en matière d’AED de 2014 à 2023.

Méthodes: Nous avons mené une étude rétrospective sur l’utilisation des médicaments à partir des fichiers libres d’accès OpenMedic du Système national d’information sanitaire (SNS). Toutes les délivrances des 24 AEDs aux enfants entre 2014 et 2023 ont été extraites par code produit. Les données annuelles d’utilisation, de conditionnement et de coûts ont été synthétisées. Les tendances temporelles ont été analysées par le test de Spearman, une régression logistique multivariée a modélisé les différences entre les sexes, la méthode LASSO a permis de sélectionner les déterminants de la délivrance de génériques.

Résultats: Au cours de la décennie, au total 2 015 504 enfants ont consommé annuellement 15 748 141 boîtes d’AED, pour un coût de 274,4 millions d’euros. Le nombre d’utilisateurs annuels a augmenté de 24 % (de 174 889 à 216 607/an). L’utilisation des AED de troisième génération a progressé de 70 % (de 98 659 utilisateurs par an à 168 290/an), tandis que celle des premières et de deuxièmes générations a diminué respectivement de 48 % (de 1 579 à 823) et de 28 % (de 87 929 à 63 038/an). L’utilisation du valproate a globalement diminué de 37 % (de 58 845 à 37 014/an) avec une baisse marquée de 62 % (-62%, de 23 480 à 8 975/an). Les prescriptions de lamotrigine et de lévétiracétam ont augmenté en parallèle de 49 % (de 29 404 à 43 847/an) et de 77 % (de 37 290 à 65 940/an). Les génériques représentaient 11,2 % des délivrances en 2014 (191 551 boîtes) contre 20,2 % en 2023 (455 934 boîtes). Les prescriptions par les psychiatres libéraux et l’utilisation de la gabapentine, de la prégabaline, de la lamotrigine et du lévétiracétam étaient des facteurs prédictifs indépendants de l’adoption des génériques. La substitution automatique par des génériques des AEDs aurait permis de réduire les dépenses de santé de 8 % en 2023 (3,88 millions d’euros).

Interprétation: En France, la prise en charge des AEDs en pédiatrie évolue rapidement vers des traitements plus sûrs et plus récents, ainsi que vers une réduction des risques liés au sexe. Cependant, la pénétration des génériques reste insuffisante. Des stratégies de substitution ciblées permettraient de dégager des fonds pour des thérapies innovantes sans compromettre le contrôle des crises.

Financement: Aucun.
