# Supplementary material for: Trends in paediatric antiseizure-medication use and costs in France, 2014–2023: a nationwide population-based analysis
Source: Lancet Reg Health Eur. 2026 Feb 3;63:101594. doi: 10.1016/j.lanepe.2026.101594 (PMC12887193; doi:10.1016/j.lanepe.2026.101594)
Supplement: Supplementary Material [file mmc2.docx]

**Title: Trends in paediatric antiseizure-medication use and costs in France, 2014-2023: a nationwide population-based analysis**

**Contents**

[Supplementary Document 1. Additional data on OpenMedic 3](#_Toc216867589)

[Supplementary Document 2. Statistical analyses 4](#_Toc216867590)

[1. Time‑trend models 4](#_Toc216867591)

[2. Multivariable logistic regression for sex differences 4](#_Toc216867592)

[3. LASSO logistic regression for generic uptake 4](#_Toc216867593)

[4. Counter‑factual cost scenario 4](#_Toc216867594)

[5. Sensitivity & robustness checks 4](#_Toc216867595)

[a. Log-linear Poisson trend for annual reimbursement (CBD excluded) 4](#_Toc216867596)

[b. Leave‑one‑year‑out re‑estimation of logistic models 5](#_Toc216867597)

[c. E‑value computation for unmeasured confounding (Valproate → Female sex) 5](#_Toc216867598)

[6. Cost analyses 5](#_Toc216867599)

[a. Describe the cost profile of each molecule in 2023 5](#_Toc216867600)

[b. Compare cost across pharmacological generations 5](#_Toc216867601)

[c. Detail Gen‑3 heterogeneity by launch decade 6](#_Toc216867602)

[d. Partition variance into “molecule” vs “generation” 6](#_Toc216867603)

[Supplementary Document 3. Estimation of ASO and gene therapy in Dravet syndrome 7](#_Toc216867604)

[1. Eligible Pediatrics Population 7](#_Toc216867605)

[2. Estimating the Number of Children with Dravet Syndrome 7](#_Toc216867606)

[3. Cost Assumptions and Dosing Regimen 7](#_Toc216867607)

[4. National Annual Cost Estimate 7](#_Toc216867608)

[Supplementary Table 1: First-, second- and third-generation antiseizure medicines approved in France (chronological order) 8](#_Toc216867609)

[Supplementary Table 2. List of CIP13 product use in this study 9](#_Toc216867610)

[Supplementary Table 3. Annual population aged < 20 years in France, 2014–2024 20](#_Toc216867611)

[Supplementary Table 4. Odds ratios for female vs male prescriptions by ASM, prescriber type, and calendar year 21](#_Toc216867612)

[Supplementary Table 5. Log-linear Gamma model for annual reimbursed expenditure on paediatric antiseizure medicines in France (excluding cannabidiol) 22](#_Toc216867613)

[Supplementary Table 6. Factors associated with generic uptake of antiseizure medications 23](#_Toc216867614)

[Supplementary Figure 1: Timeline of Market Authorization for Epilepsy-Indicated Medicines by Pharmacological Generation in France 24](#_Toc216867615)

[Supplementary Figure 2: Flowchart of Data Selection for our Study 25](#_Toc216867616)

[Supplementary Figure 3: Evolution of treatment by ASM generations between 2014 and 2023 26](#_Toc216867617)

[Supplementary Figure 4: Total Annual Pediatrics Antiepileptic Drug Consumers Over the Past Decade 27](#_Toc216867618)

[Supplementary Figure 5: Prescriber of paediatric Anti-Seizure Medications Over the Past Decade 28](#_Toc216867619)

[Supplementary Figure 6: Share of Prescriber Activity by Year 29](#_Toc216867620)

[Supplementary Figure 7: Leave-One-Year-Out Analysis Confirms Stable Association Between Valproate Exposure and Patient Sex 30](#_Toc216867621)

[Supplementary Figure 8: Annual reimbursed expenditure for paediatric antiseizure medicines in France, 2014-2023 31](#_Toc216867622)

[Supplementary Figure 9: Annual per‑patient cost of podiatric anti‑seizure medicines dispensed in 2023 32](#_Toc216867623)

[Bibliography 33](#_Toc216867624)

# Supplementary Document 1. Additional data on OpenMedic

Open Medic compiles raw data extracted from the French National Health Data System (SNDS): an exhaustive, person‐level repository that captures all claims and hospital data for ≈99% of France’s 67 million residents^1^. OpenMedic provides fully anonymised, prescription‑level records for every reimbursed medicine dispensed in community pharmacies. Each annual file lists the 13‑digit product code (CIP‑13), brand name, pack size, beneficiary sex and age‑band (0-19, 20-59, > 60 years), and prescriber speciality. All dispensing records were extracted from **Open Medic**, the open-access subset of the French National Health Data System (SNDS) that covers every community-pharmacy medicine reimbursed by national health insurance since 2014^2^.

Below is a concise definition of every field that appears in the NB_YYYY files you analysed. Use it in your Supplementary Material under the heading “Data dictionary”.

| Column | Meaning | Notes |
| --- | --- | --- |
| CIP13 | 13-digit national product identifier | Unique to each pharmaceutical presentation (brand × dosage × form). |
| L_CIP13 | Product label | Commercial name + strength and form; useful for cross-checking molecule identity. |
| AGE | Age band of the beneficiary | Three mutually exclusive groups in Open Medic: 0-19 y, 20-59 y, ≥60 y. |
| sexe | Sex of the beneficiary | “1” = male, “2” = female, “0” = unknown (very rare). |
| PSP_SPE | Prescriber speciality code | Two-digit code defined by Assurance Maladie (e.g., “01” = GP, “10” = paediatrician). |
| nbc | Consumers | Count of unique people who received at least one dispensing for that cell during the calendar year. |
| REM | Reimbursed amount (€) | Sum actually paid by national health insurance; excludes pharmacy dispensing fees from 2015 onward. |
| BSE | Health-Insurance reimbursement base | Official reference price used to calculate REM; equals REM for drugs reimbursed at 100 %, lower otherwise. |
| BOITES | Packs (boxes) dispensed | Total number of packs delivered, independent of pack size. |

We first built an exhaustive list of paediatric antiseizure-medication presentations marketed in France between 1 Jan 2014 and 31 Dec 2023 (Table S1). Using the French Medicines Agency database and annual reimbursement catalogues, we mapped every molecule and strength to its unique 13-digit product identifier (CIP-13). Twenty-four active substances met our inclusion criteria, yielding 278 distinct CIP-13 codes over the study period. CIP-13 = 340 + CIP-7 + PP + checksum. 340 anchors the code in the GS1 range reserved for French healthcare items; the CIP-7 specifies the medicinal product; the two-digit PP extension singles out a particular pack (e.g., 30-tablet box vs 60-tablet box); and the final digit ensures data integrity at scanning or electronic transmission. In Open Medic, CIP-13 uniquely identifies every reimbursed presentation and was used as the primary key for extraction and aggregation in our study.

# Supplementary Document 2. Statistical analyses

Analyses were performed with two complementary objectives: (i) describe temporal trends; (ii) identify determinants of sex‑specific prescribing and generic uptake.

## Time‑trend models

- **Spearman rank correlation (ρ)** tested monotonic trends in counts or proportions across calendar years.
- **Linear regression** estimated absolute annual changes (β̂) and 95 % CIs for normally distributed outcomes (after log‑ or square‑root transformation where appropriate). Residual normality was checked with Q-Q plots and the Shapiro-Wilk test.

## Multivariable logistic regression for sex differences

The dependent variable was *sex* (female = 1). Because Open Medic provides aggregated counts, we re‑expanded each cell into individual‑level pseudo‑records ("uncounting") so that any cell of *n* prescriptions yielded *n* rows with identical covariate values. The model specification was:

logit(P(Female)) = β0 + β1·Molecule + β2·Year (numeric) + β3·Prescriber_Type + ε

where *Molecule* and *Prescriber_Type* were entered as dummy variables (reference levels: valproate; hospital). Robust sandwich SEs were calculated (R package *sandwich^3^*) to account for residual over‑dispersion. Model fit was judged by McFadden’s R² and the Hosmer‑Lemeshow *ĉ* statistic^4,5^. Multicollinearity was assessed via the generalized variance‑inflation factor (GVIF); no GVIF exceeded 3^6^.

## LASSO logistic regression for generic uptake

To deal with multicollinearity among molecule and prescriber variables, we applied Least Absolute Shrinkage and Selection Operator (LASSO) using the *glmnet* package^7^. The outcome was *Generic (Yes = 1)* and the candidate predictors were:

- Molecule (12 binary dummies for substances with ≥1 generic),
- Prescriber_Type (6 dummies),
- Calendar year (numeric),
- Box formulation (tablet, capsule, liquid, granules).

The penalty parameter λ was selected through 10‑fold cross‑validation with the one‑standard‑error rule (λ1 se). Coefficients with absolute value <10⁻⁴ were considered shrunk to zero. We report odds ratios (ORs = e^β) with 95 % bootstrap CIs (1 000 resamples).

## Counter‑factual cost scenario

Observed 2023 spend (= Σ boxes unit_price × units) was compared with a scenario in which each originator box was repriced at the mean reimbursed price of its bio‑equivalent generics. The difference represents potential savings. Uncertainty was propagated via a non‑parametric bootstrap (2 000 iterations) drawing at the molecule‑year level.

## Sensitivity & robustness checks

- **Negative‑binomial regression** for over‑dispersed count outcomes (annual users, boxes)
- **Poisson log‑linear trend**^8^ after excluding cannabidiol (2023 market entry) to confirm robustness of cost plateau
- **Leave‑one‑year‑out** re‑estimation of logistic models^9^
- **E‑value**^10^ computation to quantify unmeasured confounding for the sex-valproate association

### Log-linear Poisson trend for annual reimbursement (CBD excluded)

**Objective.** Confirm that the apparent plateau in total ASM costs after 2020 is not an artefact of cannabidiol’s 2023 market entry.

**Model specification.** Poisson log-linear model on annual reimbursements (REM) aggregated by calendar year **after exclusion of all CBD/CANNABIDIOL dispensing**; year centred at 2014:

log⁡(μt)=β0+β1(Yeart−2014),Yt∼Poisson(μt).\log(\mu_t)=\beta_0+\beta_1(\text{Year}_t-2014),\quad Y_t\sim\text{Poisson}(\mu_t).log(μt​)=β0​+β1​(Yeart​−2014),Yt​∼Poisson(μt​).

### Leave‑one‑year‑out re‑estimation of logistic models

**Objective.** Test whether the association between valproate exposure and patient sex is driven by any single calendar year.

**Model.** Weighted logistic regression (glm, family = binomial) fitted on individual dispensing with weights = nbc (number of paediatric beneficiaries per record). The model included valproate exposure (binary), prescriber type and a natural cubic spline for calendar year.

**Leave‑one‑year‑out (LOYO) results.** Removing each year 2014‑2023 in turn yields very similar odds ratios. The odds ratio remains between 0.41 and 0.45 with overlapping confidence intervals, indicating that no single year materially influences the sex‑specific prescribing pattern.

**Interpretation.** Valproate dispensing are consistently ~58 % less likely to be made to female patients compared with other ASMs across the entire study period. This robustness supports the primary finding and mitigates concerns of period‑specific confounding.

Removing 2017 raises the OR from 0.442 to 0.449, consistent with the legislative tightening of March 2017 that curtailed female valproate prescriptions. Nonetheless, the association remains stable (range 0.417-0.449), confirming that no single year overturns the finding.

### E‑value computation for unmeasured confounding (Valproate → Female sex)

**Objective.** Quantify the minimum strength of an unmeasured confounder that would be required to explain away the observed association between valproate exposure and female sex.

**Method.** Using the point estimate from the weighted logistic model (OR ≈ 0.44), we converted it to a risk ratio (RR ≈ 0.666) because sex is not a rare outcome (> 40 %). The EValue package (version 3.x) was applied with evalues.OR(…, rare = FALSE).

| Effect measure | Point estimate | 95 % CI | E‑value (point) | E‑value (upper CI) |
| --- | --- | --- | --- | --- |
| Risk ratio (Female vs Male) | 0.666 | 0.664 - 0.668 | 2.37 | 2.36 |

**Interpretation.** An unmeasured confounder would need to increase *both* the odds of being dispensed valproate *and* the odds of being female by at least 2.3‑fold each to fully negate the association. Given that the model already adjusts for prescriber type and calendar year, such a strong confounder is unlikely, supporting the robustness of our findings.

## Cost analyses

### Describe the cost profile of each molecule in 2023

| ASM | Generation | Launched | Annual cost per individual (euros) |
| --- | --- | --- | --- |
| PB | 1 | - | 11,5 [7,6-17,3] |
| PRM | 1 | - | 36,8 [26,9-37,4] |
| PHT | 1 | - | 48,5 [41,1-55,4] |
| CBZ | 2 | - | 16 [8,6-25,5] |
| VPA | 2 | - | 45,8 [30,8-67,9] |
| ESM | 2 | - | 53,9 [50,2-65,8] |
| GBP | 3 | 2000 | 30 [17,7-44,1] |
| LTG | 3 | 2000 | 30,1 [19,8-40,9] |
| TPM | 3 | 2000 | 38,9 [27,2-66,9] |
| OXC | 3 | 2000 | 64,7 [39,3-128,8] |
| VGB | 3 | 2000 | 252,6 [214-380,3] |
| PGB | 3 | 2010 | 20,4 [12-39,9] |
| ZNS | 3 | 2010 | 45,8 [28,8-71,8] |
| LEV | 3 | 2010 | 77,4 [58,4-113,1] |
| LCM | 3 | 2010 | 93,8 [55,3-250] |
| RFM | 3 | 2010 | 538,7 [316,6-887,4] |
| ESL | 3 | 2010 | 594,9 [528,8-646,9] |
| STP | 3 | 2010 | 1570,9 [1303,6-2348,2] |
| CNB | 3 | 2020 | 143,9 [102,7-193,2] |
| BRV | 3 | 2020 | 321,4 [293,5-401,2] |
| PER | 3 | 2020 | 350,9 [277,3-493,1] |
| FFA | 3 | 2020 | 5787,7 [3188,1-6603,7] |
| CBD | 3 | 2020 | 10272 [6041,7-14396,6] |
| EVER | 3 | 2020 | 15583,9 [9151,6-25865,9] |

### Compare cost across pharmacological generations

A monotonic increase in per‑patient annual cost across pharmacological generations was confirmed by a Jonckheere-Terpstra trend test^11^ (JT = 178 133; two‑sided p = 3·1 × 10⁻⁸, normal approximation, n = 1 467).

To quantify the magnitude of this trend we fitted an ordinary‑least‑squares model to log‑transformed costs, specifying log Cost = β₀ + β₁·GenCode (GenCode = 1, 2, 3). The slope was β₁ = 0·509 with a standard error of 0·089 (t = 5·73, p = 1·1 × 10⁻⁸). Back‑transformation shows that each step from Gen 1 to Gen 2 or from Gen 2 to Gen 3 multiplies the median annual cost by 1·66 (95 % CI 1·41-1·92), i.e., a 66 % increase per generation. Residuals were approximately normal (Shapiro-Wilk p = 0·14) with no evidence of heteroscedasticity (Breusch-Pagan p = 0·21), indicating an adequate log‑linear fit.

In short, costs rise steeply and consistently with pharmacological generation, with each generational step adding roughly two‑thirds to the median annual expenditure per patient.

### Detail Gen‑3 heterogeneity by launch decade

Within Generation 3, costs varied strongly with the decade in which each molecule was first licensed. Median annual cost per patient rose from €72 [IQR 29-148] for molecules launched in the 1990s, to €215 [IQR 115-442] for those launched in the 2000s, and reached €1 545 [IQR 238-6 604] for molecules introduced since 2010.

A Jonckheere-Terpstra trend test confirmed a highly significant monotonic increase across these three decades (JT = 302 807, p < 2 × 10⁻¹⁶). Post‑hoc Wilcoxon comparisons, Bonferroni‑adjusted for the three pairings, showed that every step was significant: 1990s vs 2000s (p ≈ 7 × 10⁻¹⁹), 1990s vs 2010s (p ≈ 4 × 10⁻⁴⁴), and 2000s vs 2010s (p ≈ 7 × 10⁻³¹). Thus, even within the third pharmacological generation, each successive decade of market entry is associated with a substantial—and statistically robust—rise in per‑patient expenditure.

### Partition variance into “molecule” vs “generation”

After variability due to individual molecules was modelled as a random intercept, pharmacological generation remained an independent cost driver.

In the mixed‑effects model log (cost) = β₀ + β₁ · Generation (1 → 2 → 3) + (1 | Molecule), the generation slope was β₁ = 1.31 ± 0.53 (t = 2.46; p = 0.014). Back‑transforming gives a 3.7‑fold increase in median annual cost for each step up the generational ladder (95 % CI 1.3 - 10.5).

Variance partitioning showed that most heterogeneity lay between molecules: the random intercept for molecule had a variance of 3.23 (SD = 1.80), whereas the within‑molecule residual variance was only 0.44 (SD = 0.66). Accordingly, fixed effects (generation alone) explained 7.7 % of the total variance (marginal R² = 0.08), while the full mixed model captured 89 % (conditional R² = 0.89).

Interpretation. Which ASM is prescribed is the primary determinant of cost, but moving from one pharmacological generation to the next still multiplies per‑patient expenditure by nearly fourfold, independent of molecule‑specific price differences.

# Supplementary Document 3. Estimation of ASO and gene therapy in Dravet syndrome

To approximate the financial impact of introducing both antisense oligonucleotide (ASO) therapy (zorevunersen) and gene therapy for Dravet syndrome (DS) in France, we used a population-based model grounded in national birth records, incidence estimates, and treatment pricing proxies.

## Eligible Pediatrics Population

We defined the eligible population for ASO therapy as all individuals aged 1 to 19 years in 2023, excluding infants under one year, as DS is typically diagnosed around the age of one in France. Based on official birth records from INSEE, we calculated the number of live births in France between 2004 and 2022, yielding a total of 15,893,343 children potentially within this age group.

For the gene therapy scenario, we restricted eligibility to children aged 1 to 2 years, the presumed optimal window for early genetic intervention. According to national birth statistics, this subgroup includes 725,997 children born in 2022^12^.

## Estimating the Number of Children with Dravet Syndrome

Based on recent systematic reviews of DS epidemiology, incidence estimates range between **2.2 and 6.5 per 100,000 live births^13^.** We adopted the median incidence rate of 4.35 per 100,000, providing a conservative yet balanced approximation. For ages 1-19, this results in an estimated ~690 individuals with DS in 2023 and for the 1-2-year age group, ~32 newly diagnosed children. These modelled values are consistent with data from the French National Rare Disease Registry (BNDMR), which reported 847 living individuals with DS, including adults (Rapport-BNDMR-20241203.pdf), supporting the validity of our estimates^14^.

## Cost Assumptions and Dosing Regimen

For zorevunersen, since pricing has not been finalized, we used the cost of nusinersen (Spinraza) as a proxy, currently priced at €63,175 per dose in France^15^. Unlike nusinersen's intensive loading schedule, zorevunersen is expected to require three injections per year (one every four months), based on the Phase 3 EMPOWER trial by Stoke Therapeutics^16^.

Thus, the annual treatment cost per patient would be approximately: €63 175× 3 = €189 525 per year per patient

For gene therapy, we used the pricing of onasemnogene abeparvovec (Zolgensma) as a benchmark, currently among the most expensive approved gene therapies globally, at €1,945,000 per patient, delivered as a single one-time dose^17^.

## National Annual Cost Estimate

ASO therapy (ages 1-19): Multiplying the estimated paediatric individual count by annual treatment cost: **690 patients × €189 525= €** **130 772 250 /year**

Gene therapy (ages 1): 62 patients × €1,945,000 = €6,224,000 /year (one-time cost)

# Supplementary Table 1: First-, second- and third-generation antiseizure medicines approved in France (chronological order****)****

Launch years correspond to the French marketing authorisation reported in the SmPCs for epilepsy. 5-HT₂ = 5-hydroxytryptamine type-2 (serotonin) receptor; ADD-ON = adjunctive therapy; ADR = adverse drug reaction; AMPA = α-amino-3-hydroxy-5-methyl-4-isoxazole-propionic-acid receptor; Ca²⁺ = calcium ion; Cl⁻ = chloride ion; DRESS = drug reaction with eosinophilia and systemic symptoms; GABA = γ-aminobutyric acid; K⁺ = potassium ion; LGS = Lennox-Gastaut syndrome; mTOR = mechanistic (mammalian) target of rapamycin; Na⁺ = sodium ion; NMDA = N-methyl-D-aspartate receptor; PR = atrioventricular PR interval on the ECG; QT = ventricular repolarisation interval on the ECG; SJS = Stevens-Johnson syndrome; SV2A = synaptic-vesicle protein 2A; TSC = tuberous-sclerosis complex; ↑ = increase. “Y” = generics available; “N” = originator only.

| Molecule | French launch year | paediatric licence (age / indication) | Generics | Mechanism of action (simplified) | Main adverse effects (selected) |
| --- | --- | --- | --- | --- | --- |
| Phenobarbital | 1920 | From birth - all epilepsies - mono | Y | ↑ brain GABA concentration | Agitation, hyperactivity, sleep disturbance; somnolence/instability at high doses |
| Phenytoin | 1941 | From birth - all epilepsies - mono | N | Voltage-dependent Ca²⁺-channel block | Gingival hypertrophy (preventable with dental hygiene) |
| Primidone | 1953 | From birth - all epilepsies - mono | N | Cl⁻-channel opening; membrane stabilization | Somnolence, vertigo, rash, mood changes |
| Ethosuximide | 1962 | ≥3 y - generalized epilepsy - mono | N | Ca²⁺-channel block | Nausea, anorexia; somnolence at high dose |
| Carbamazepine | 1964 | From birth - focal epilepsy - mono | Y | Voltage-dependent Na⁺-channel block | Somnolence, diplopia, rash (may require discontinuation) |
| Valproic acid | 1967 | From birth - all epilepsies | Y | ↑ brain GABA; Ca²⁺-channel block | weight gain, hair loss (mild), tremor, hepatotoxicity, teratogenicity |
| Vigabatrin | 1991 | Infantile spasms mono; focal add-on | N | Irreversible GABA-transaminase inhibition | Visual-field loss, weight gain, myoclonus |
| Felbamate | 1994 | ≥4 y - Lennox-Gastaut - add-on | N | NMDA-receptor blockade | Rash, aplastic anaemia, hepatic failure |
| Gabapentin | 1995 | ≥6 y add-on; ≥12 y mono - focal epilepsy | Y | GABA analogue ; Ca²⁺-Channel block | Ataxia, somnolence, behavioural disorders in encephalopathic children |
| Lamotrigine | 1996 | ≥2 y add-on; ≥13 y mono - all epilepsies | Y | Na⁺-channel block | Rash (risk of SJS), headache, tremor if titrated too fast |
| Tiagabine | 1996 | ≥12 y add-on - focal epilepsy | N | ↑ brain GABA | Dizziness, nervousness |
| Topiramate | 1998 | ≥2 y add-on / ≥6 y mono - all epilepsies | Y | Ca²⁺-channel block; NMDA inhibition | Weight loss, cognitive slowing, renal stones |
| Oxcarbazepine | 2000 | ≥6 y mono - focal epilepsy | Y | Na⁺-channel block | Similar to carbamazepine but milder |
| Levetiracetam | 2002 | ≥1 mo. add-on (focal); ≥16 y mono (focal); ≥12 y add-on (generalized) | Y | SV2A modulation (GABA-A/ Ca²⁺) | Somnolence; minimal interactions |
| Pregabalin | 2004 | >18 y add-on - focal epilepsy | Y | Ca²⁺-channel block | Weight gain, dizziness, blurred vision |
| Zonisamide | 2005 | ≥6 y add-on; >18 y mono - focal epilepsy | Y | Voltage-dependent Na⁺- and Ca²⁺-channel block | Anorexia, agitation/irritability, kidney stones, DRESS |
| Rufinamide | 2007 | ≥1 y - Lennox-Gastaut - add-on | N | Na⁺-channel block | Dizziness, QT-shortening caution |
| Stiripentol | 2007 | ≥3 y - add-on for generalized epilepsy | N | ↑ brain GABA | CYP450 inhibition; dose reduction of co-meds |
| Lacosamide | 2008 | ≥2 y mono (focal); ≥4 y add-on (generalized) | Y | Voltage-dependent Na⁺-channel block | Dizziness, diplopia, PR prolongation |
| Eslicarbazepine | 2009 | >18 y mono; ≥6 y add-on - focal epilepsy | N | Voltage-dependent Na⁺-channel block | Nausea, vertigo, serious skin reactions |
| Perampanel | 2011 | ≥4 y add-on (focal); ≥7 y add-on (generalized) | N | AMPA-receptor antagonist | Dizziness, aggression |
| Retigabine | 2012, withdrawn 2017 | >18 y add-on - focal epilepsy | N | K⁺-channel opener | Withdrawn for pigment changes |
| Brivaracetam | 2016 | ≥2 y - focal epilepsy - add-on | N | Voltage-dependent Na⁺-channel block | Dizziness, somnolence, headache |
| Everolimus | 2018 | ≥2 y - tuberous-sclerosis complex epilepsy - add-on | N | mTOR pathway inhibition | Stomatitis, diarrhoea |
| Cannabidiol | 2019 | ≥2 y - Dravet / Lennox-Gastaut (with clobazam); TSC - add-on | N | Intracellular Ca²⁺ modulation; adenosine re-uptake inhibition | Transaminase elevation, weight loss, suicidal ideation monitoring |
| Fenfluramine | 2021 | ≥2 y - Dravet - add-on | N | 5-HT₂ receptor agonist | Weight loss, behavioural changes, cardiovascular risk |
| Cenobamate | 2021 | >18 y - focal epilepsy - 3rd-line add-on | N | Na⁺-channel inactivation & persistent current inhibition | Psychiatric ADRs (suicidality), DRESS |

| Supplementary Table 2. List of CIP13 product use in this study | | | | | |
| --- | --- | --- | --- | --- | --- |
|  |  |  |  |  |  |
| ASM | generic/princeps | CIP13 | l_cip13 |  |  |
| BRV | Princeps | 3400930053621 | BRIVIACT 10MG, CPR 14 | |  |
|  |  | 3400930053645 | BRIVIACT 25MG CPR 56 | |  |
|  |  | 3400930053652 | BRIVIACT 50MG, CPR 56 | |  |
|  |  | 3400930053669 | BRIVIACT 75MG, CPR 56 | |  |
|  |  | 3400930053676 | BRIVIACT 100MG CPR 56 | |  |
|  |  | 3400930053683 | BRIVIACT 10MG/ML, SOLUTION BUVABLE 1 | | |
| CBD | Princeps | 3400930187968 | EPIDYOLEX 100MG/ML SOL BUV 100ML 1 | | |
| CBZ | Princeps | 3400931035459 | TEGRETOL 200MG CPR 50 | |  |
|  |  | 3400932956395 | TEGRETOL 20MG/ML SUSP BUV FL 150ML 1 | | |
|  |  | 3400932984312 | TEGRETOL LP 400MG CPR 30 | | |
|  |  | 3400933148744 | TEGRETOL LP 200MG CPR 30 | | |
|  | Generic | 3400934436376 | CARBAMAZEPINE SDZ LP 200MG CPR 30 | | |
|  |  | 3400934436895 | CARBAMAZEPINE SDZ LP 400MG CPR 30 | | |
|  |  | 3400934837371 | CARBAMAZEPINE TVC 200MG CPR 50 | | |
|  |  | 3400935305145 | CARBAMAZEPINE MYL LP 400MG CPR 30 | | |
|  |  | 3400935305145 | CARBAMAZEPINE VIATRIS LP 400MG CPR 30 | | |
|  |  | 3400935306326 | CARBAMAZEPINE MYL LP 200MG CPR 30 | | |
|  |  | 3400935306326 | CARBAMAZEPINE VIATRIS LP 200MG CPR 30 | | |
| CNB | Princeps | 3400930230039 | ONTOZRY 12,5 + 25MG CPR 28 | | |
|  |  | 3400930230046 | ONTOZRY 50MG CPR 14 | |  |
|  |  | 3400930230053 | ONTOZRY 50MG CPR 28 | |  |
|  |  | 3400930230060 | ONTOZRY 100MG CPR 14 | |  |
|  |  | 3400930230084 | ONTOZRY 100MG CPR 28 | |  |
|  |  | 3400930230091 | ONTOZRY 150MG CPR 14 | |  |
|  |  | 3400930230107 | ONTOZRY 150MG CPR 28 | |  |
|  |  | 3400930230121 | ONTOZRY 200MG CPR 14 | |  |
|  |  | 3400930230145 | ONTOZRY 200MG CPR 28 | |  |
| ESL | Princeps | 3400939734996 | ZEBINIX 800MG CPR 30 | |  |
| ESM | Princeps | 3400932507955 | ZARONTIN 250MG/5ML SIROP 200ML 1 | | |
| EVER | Princeps | 3400921947588 | VOTUBIA 2,5MG CPR 30 | |  |
|  |  | 3400921947649 | VOTUBIA 5MG CPR 30 | |  |
|  |  | 3400921947700 | VOTUBIA 10MG CPR 30 | |  |
|  |  | 3400927643514 | VOTUBIA 2MG CPR DISP 30 | | |
|  |  | 3400927643682 | VOTUBIA 3MG CPR DISP 30 | | |
|  |  | 3400927643743 | VOTUBIA 5MG CPR DISP 30 | | |
|  | Generic | 3400930152423 | EVEROLIMUS MYL 5MG CPR 30 | | |
|  |  | 3400930152690 | EVEROLIMUS BGA 5MG CPR 30 | | |
| FFA | Princeps | 3400930224359 | FINTEPLA 2,2MG/ML F60ML+S3ML 1 | | |
|  |  | 3400930224366 | FINTEPLA 2,2MG/ML 120ML+S3ML 1 | | |
|  |  | 3400930224373 | FINTEPLA 2,2MG/ML 360ML+S6ML 1 | | |
| GBP | Princeps | 3400933789862 | NEURONTIN 100MG GELULE 90 | | |
|  |  | 3400933790172 | NEURONTIN 300MG GELULE 90 | | |
|  |  | 3400933801441 | NEURONTIN 400MG GELULE 90 | | |
|  |  | 3400935900258 | NEURONTIN 600MG CPR 90 | | |
|  |  | 3400935900487 | NEURONTIN 800MG CPR 90 | | |
|  | Generic | 3400922032108 | GABAPENTINE SDZ 600MG CPR 90 | | |
|  |  | 3400926742638 | GABAPENTINE ARG 600MG CPR 90 | | |
|  |  | 3400927481970 | GABAPENTINE PFZ 100MG GELULE 90 | | |
|  |  | 3400927482052 | GABAPENTINE PFZ 300MG GELULE 90 | | |
|  |  | 3400927482113 | GABAPENTINE PFZ 400MG GELULE 90 | | |
|  |  | 3400927482342 | GABAPENTINE PFZ 600MG CPR 90 | | |
|  |  | 3400930064665 | GABAPENTINE BGA 100MG GELULE 90 | | |
|  |  | 3400930064672 | GABAPENTINE BGA 300MG GELULE 90 | | |
|  |  | 3400930082454 | GABAPENTINE TVS 600MG CPR 90 | | |
|  |  | 3400930082508 | GABAPENTINE TVS 800MG CPR 90 | | |
|  |  | 3400930149225 | GABAPENTINE ZYD 100MG GELULE 90 | | |
|  |  | 3400930149263 | GABAPENTINE ZYD 300MG GELULE 90 | | |
|  |  | 3400930169452 | GABAPENTINE VIATRIS 600MG CPR 90 | | |
|  |  | 3400930169476 | GABAPENTINE VTS 800MG CPR 90 | | |
|  |  | 3400936613577 | GABAPENTINE TVC 600MG CPR 90 | | |
|  |  | 3400936677852 | GABAPENTINE TVC 100MG GELULE 90 | | |
|  |  | 3400936678514 | GABAPENTINE TVC 300MG GELULE 90 | | |
|  |  | 3400936678743 | GABAPENTINE TVC 400MG GELULE 90 | | |
|  |  | 3400936679054 | GABAPENTINE ARW 400MG GELULE 90 | | |
|  |  | 3400936680463 | GABAPENTINE BGA 400MG GELULE 90 | | |
|  |  | 3400936681293 | GABAPENTINE MYL 400MG GELULE 90 | | |
|  |  | 3400936681293 | GABAPENTINE VIATRIS 400MG GELULE 90 | | |
|  |  | 3400936942103 | GABAPENTINE ZEN 400MG GELULE 90 | | |
|  |  | 3400936942103 | GABAPENTINE ZTL 400MG GELULE 90 | | |
|  |  | 3400937411394 | GABAPENTINE SDZ 300MG GELULE 90 | | |
|  |  | 3400937411455 | GABAPENTINE SDZ 100MG GELULE 90 | | |
|  |  | 3400937441902 | GABAPENTINE EVO 300MG GELULE 90 | | |
|  |  | 3400937442442 | GABAPENTINE BGA 300MG GELULE 90 | | |
|  |  | 3400937443043 | GABAPENTINE BGA 100MG GELULE 90 | | |
|  |  | 3400937443791 | GABAPENTINE ZEN 100MG GELULE 90 | | |
|  |  | 3400937443791 | GABAPENTINE ZTL 100MG GELULE 90 | | |
|  |  | 3400937444453 | GABAPENTINE ZEN 300MG GELULE 90 | | |
|  |  | 3400937444453 | GABAPENTINE ZTL 300MG GELULE 90 | | |
|  |  | 3400937446693 | GABAPENTINE EVO 100MG GELULE 90 | | |
|  |  | 3400937521666 | GABAPENTINE ARW 100MG GELULE 90 | | |
|  |  | 3400937523158 | GABAPENTINE ARW 300MG GELULE 90 | | |
|  |  | 3400937666428 | GABAPENTINE SDZ 400MG GELULE 90 | | |
|  |  | 3400937729505 | GABAPENTINE MYL 800MG CPR 90 | | |
|  |  | 3400937734066 | GABAPENTINE MYL 600MG CPR 90 | | |
|  |  | 3400937788236 | GABAPENTINE BGA 600MG CPR 90 | | |
|  |  | 3400937788984 | GABAPENTINE BGA 800MG CPR 90 | | |
|  |  | 3400937789875 | GABAPENTINE MYL 100MG GELULE 90 | | |
|  |  | 3400937789875 | GABAPENTINE VIATRIS 100MG GELULE 90 | | |
|  |  | 3400937791137 | GABAPENTINE MYL 300MG GELULE 90 | | |
|  |  | 3400937791137 | GABAPENTINE VIATRIS 300MG GELULE 90 | | |
|  |  | 3400937792486 | GABAPENTINE ZEN 600MG CPR 90 | | |
|  |  | 3400937792486 | GABAPENTINE ZTL 600MG CPR 90 | | |
|  |  | 3400937970518 | GABAPENTINE ARW 800MG CPR 90 | | |
|  |  | 3400937970747 | GABAPENTINE ARW 600MG CPR 90 | | |
|  |  | 3400938002157 | GABAPENTINE EGL 100MG GELULE 90 | | |
|  |  | 3400938006810 | GABAPENTINE EGL 300MG GELULE 90 | | |
|  |  | 3400938161793 | GABAPENTINE EGL 400MG GELULE 90 | | |
|  |  | 3400939203799 | GABAPENTINE EGL 600MG CPR 90 | | |
|  |  | 3400949921744 | GABAPENTINE ARG 100MG GELULE 90 | | |
|  |  | 3400949922574 | GABAPENTINE ARG 300MG GELULE 90 | | |
|  |  | 3400949924585 | GABAPENTINE ARG 400MG GELULE 90 | | |
| LCM | Princeps | 3400922198149 | VIMPAT 10MG/ML SIR FL 200ML 1 | | |
|  |  | 3400938829570 | VIMPAT 50MG CPR 14 | |  |
|  |  | 3400938829631 | VIMPAT 50MG CPR 56 | |  |
|  |  | 3400938829921 | VIMPAT 100MG CPR 56 | |  |
|  |  | 3400938830170 | VIMPAT 150MG CPR 56 | |  |
|  |  | 3400938830460 | VIMPAT 200MG CPR 56 | |  |
|  | Generic | 3400930126172 | LACOSAMIDE MEDIPHA 50MG CPR 14 | | |
|  |  | 3400930126196 | LACOSAMIDE MEDIPHA 50MG CPR 56 | | |
|  |  | 3400930126219 | LACOSAMIDE MEDIPHA 100MG CPR 56 | | |
|  |  | 3400930126233 | LACOSAMIDE MEDIPHA 150MG CPR 56 | | |
|  |  | 3400930126264 | LACOSAMIDE MEDIPHA 200MG CPR 56 | | |
|  |  | 3400930174074 | LACOSAMIDE EG 50MG CPR 56 | | |
|  |  | 3400930174173 | LACOSAMIDE EG 100MG CPR 56 | | |
|  |  | 3400930189801 | LACOSAMIDE SDZ 50MG CPR 56 | | |
|  |  | 3400930189856 | LACOSAMIDE SDZ 100MG CPR 56 | | |
|  |  | 3400930189917 | LACOSAMIDE SDZ 150MG CPR 56 | | |
|  |  | 3400930189986 | LACOSAMIDE SDZ 200MG CPR 56 | | |
|  |  | 3400930213674 | LACOSAMIDE ZENTIVA 50MG CPR 56 | | |
|  |  | 3400930213698 | LACOSAMIDE ZENTIVA 100MG CPR 56 | | |
|  |  | 3400930213735 | LACOSAMIDE ZENTIVA 200MG CPR 56 | | |
|  |  | 3400930229637 | LACOSAMIDE ARW 100MG CPR PEL SEC 56 | | |
|  |  | 3400930229651 | LACOSAMIDE ARW 200MG CPR PEL SEC 56 | | |
|  |  | 3400930247075 | LACOSAMIDE VIATRIS 50MG CPR 56 | | |
|  |  | 3400930247082 | LACOSAMIDE VIATRIS 100MG CPR 56 | | |
|  |  | 3400930247099 | LACOSAMIDE VIATRIS 150MG CPR 56 | | |
|  |  | 3400930247143 | LACOSAMIDE VIATRIS 200MG CPR 56 | | |
|  |  | 3400930247327 | LACOSAMIDE TEVA 50MG CPR 56 | | |
|  |  | 3400930247334 | LACOSAMIDE TEVA 100MG CPR 56 | | |
|  |  | 3400930247341 | LACOSAMIDE TEVA 150MG CPR 56 | | |
|  |  | 3400930247358 | LACOSAMIDE TEVA 200MG CPR 56 | | |
| LEV | Princeps | 3400927996979 | LEVIDCEN 250MG GRANULES SAC 60 | | |
|  |  | 3400927997570 | LEVIDCEN 500MG GRANULES SAC 60 | | |
|  |  | 3400927998232 | LEVIDCEN 750MG GRANULES SAC 60 | | |
|  |  | 3400927998980 | LEVIDCEN 1000MG GRANULES SAC 60 | | |
|  |  | 3400935601360 | KEPPRA 250MG CPR 60 | |  |
|  |  | 3400935601650 | KEPPRA 500MG CPR 60 | |  |
|  |  | 3400935602251 | KEPPRA 1000MG CPR 60 | |  |
|  |  | 3400937023818 | KEPPRA 100MG/ML SOL BUV 300ML+S10ML 1 | | |
|  |  | 3400939827599 | KEPPRA 100MG/ML SOL BUV 150ML+S3ML 1 | | |
|  |  | 3400939827650 | KEPPRA 100MG/ML SOL BUV 150ML+S1ML 1 | | |
|  |  | 3400949504831 | KEPPRA 1000MG CPR 60 | |  |
|  |  | 3400949505081 | KEPPRA 500MG CPR 60 | |  |
|  |  | 3400949508914 | KEPPRA 500MG CPR 60 | |  |
|  |  | 3400949510405 | KEPPRA 500MG CPR 60 | |  |
|  |  | 3400949510634 | KEPPRA 500MG CPR 60 | |  |
|  |  | 3400949510863 | KEPPRA 1000MG CPR 60 | |  |
|  |  | 3400949512294 | KEPPRA 250MG CPR 60 | |  |
|  | Generic | 3400921625929 | LEVETIRACETAM TVC 250MG CPR 60 | | |
|  |  | 3400921626001 | LEVETIRACETAM TVC 500MG CPR 60 | | |
|  |  | 3400921626179 | LEVETIRACETAM TVC 1000MG CPR 60 | | |
|  |  | 3400921923285 | LEVETIRACETAM ACTAVIS 250MG CPR 60 | | |
|  |  | 3400921923346 | LEVETIRACETAM ACTAVIS 500MG CPR 60 | | |
|  |  | 3400921925647 | LEVETIRACETAM SDZ 250MG CPR 60 | | |
|  |  | 3400921926767 | LEVETIRACETAM SDZ 500MG CPR 60 | | |
|  |  | 3400921927719 | LEVETIRACETAM SDZ 1000MG CPR 60 | | |
|  |  | 3400921939255 | LEVETIRACETAM SDZ 100MG/ML SOL BUV 1 | | |
|  |  | 3400922030616 | LEVETIRACETAM ARW 500MG CPR 60 | | |
|  |  | 3400922031217 | LEVETIRACETAM ARW 250MG CPR 60 | | |
|  |  | 3400922135854 | LEVETIRACETAM ARW 1000MG CPR 60 | | |
|  |  | 3400922325941 | LEVETIRACETAM RBX 500MG CPR 60 | | |
|  |  | 3400922381602 | LEVETIRACETAM ACC 500MG CPR 60 | | |
|  |  | 3400926610364 | LEVETIRACETAM ZTL 250MG CPR 60 | | |
|  |  | 3400926610883 | LEVETIRACETAM ZTL 500MG CPR 60 | | |
|  |  | 3400926612085 | LEVETIRACETAM ZTL 1000MG CPR 60 | | |
|  |  | 3400926803063 | LEVETIRACETAM ARW SOL BUV150ML+S1ML 1 | | |
|  |  | 3400926803124 | LEVETIRACETAM ARW SOL BUV150ML+S3ML 1 | | |
|  |  | 3400926803292 | LEVETIRACETAM ARW SOL BUV300ML+S10ML 1 | | |
|  |  | 3400926803933 | LEVETIRACETAM ARL 250MG CPR 60 | | |
|  |  | 3400926803933 | LEVETIRACETAM ARW 250MG CPR 60 | | |
|  |  | 3400926804305 | LEVETIRACETAM ARL 500MG CPR 60 | | |
|  |  | 3400926804305 | LEVETIRACETAM ARW 500MG CPR 60 | | |
|  |  | 3400926805364 | LEVETIRACETAM ARL 1000MG CPR 60 | | |
|  |  | 3400926805364 | LEVETIRACETAM ARW 1000MG CPR 60 | | |
|  |  | 3400926807894 | LEVETIRACETAM ARL 500MG CPR 30 | | |
|  |  | 3400926807894 | LEVETIRACETAM ARW 500MG CPR 30 | | |
|  |  | 3400926851897 | LEVETIRACETAM MYP SOL BUV 300ML 1 | | |
|  |  | 3400926851897 | LEVETIRACETAM VIATRIS SOL BUV 300ML 1 | | |
|  |  | 3400926852559 | LEVETIRACETAM AVG 100MG/ML SOL BUV 1/300 ML | | |
|  |  | 3400926852610 | LEVETIRACETAM AVG100MG/ML SOL BUV 1/300 ML | | |
|  |  | 3400926852788 | LEVETIRACETAM AVG100MG/ML SOL BUV 1/300 ML | | |
|  |  | 3400927447914 | LEVETIRACETAM EG 250MG CPR 60 | | |
|  |  | 3400927448393 | LEVETIRACETAM EG 500MG CPR 60 | | |
|  |  | 3400927449574 | LEVETIRACETAM EG 1000MG CPR 60 | | |
|  |  | 3400927498190 | LEVETIRACETAM ZYD 250MG CPR 60 | | |
|  |  | 3400927498541 | LEVETIRACETAM ZYD 500MG CPR 60 | | |
|  |  | 3400927646645 | LEVETIRACETAM ACTAVIS 250MG CPR 60 | | |
|  |  | 3400927646706 | LEVETIRACETAM ACTAVIS 500MG CPR 60 | | |
|  |  | 3400927646874 | LEVETIRACETAM ACTAVIS 1000MG CPR 60 | | |
|  |  | 3400927802492 | LEVETIRACETAM CRT 250MG CPR 60 | | |
|  |  | 3400927803215 | LEVETIRACETAM CRT 500MG CPR 60 | | |
|  |  | 3400927805455 | LEVETIRACETAM CRT 1000MG CPR 60 | | |
|  |  | 3400930004104 | LEVETIRACETAM KRK 500MG CPR 60 | | |
|  |  | 3400930017852 | LEVETIRACETAM EVO 500MG CPR 60 | | |
|  |  | 3400930076385 | LEVETIRACETAM ZEN 250MG CPR 60 | | |
|  |  | 3400930076385 | LEVETIRACETAM ZTL 250MG CPR 60 | | |
|  |  | 3400930076439 | LEVETIRACETAM ZEN 500MG CPR 60 | | |
|  |  | 3400930076439 | LEVETIRACETAM ZTL 500MG CPR 60 | | |
|  |  | 3400930076507 | LEVETIRACETAM ZEN 1 000MG CPR 60 | | |
|  |  | 3400930076507 | LEVETIRACETAM ZTL 1 000MG CPR 60 | | |
|  |  | 3400930080061 | LEVETIRACETAM BGA 250MG CPR 60 | | |
|  |  | 3400930080078 | LEVETIRACETAM BGA 500MG CPR 60 | | |
|  |  | 3400930151167 | LEVETIRACETAM MYP 500MG CPR 60 | | |
|  |  | 3400930151167 | LEVETIRACETAM VIATRIS 500MG CPR 60 | | |
|  |  | 3400930158463 | LEVETIRACETAM EG SOL BUV300ML+S10ML 1 | | |
|  |  | 3400941626104 | LEVETIRACETAM MYP 250MG CPR 60 | | |
|  |  | 3400941626104 | LEVETIRACETAM VIATRIS 250MG CPR 60 | | |
|  |  | 3400941626913 | LEVETIRACETAM MYP 500MG CPR 60 | | |
|  |  | 3400941627224 | LEVETIRACETAM MYP 1000MG CPR 60 | | |
|  |  | 3400941627224 | LEVETIRACETAM VIATRIS 1000MG CPR 60 | | |
|  |  | 3400941656378 | LEVETIRACETAM BGA 500MG CPR 60 | | |
|  |  | 3400941656729 | LEVETIRACETAM BGA 1000MG CPR 60 | | |
|  |  | 3400941656958 | LEVETIRACETAM BGA 250MG CPR 60 | | |
| LTG | Princeps | 3400930186350 | LAMICTAL 5MG CPR DISP 30 | | |
|  |  | 3400933898434 | LAMICTAL 25MG CPR DISP +S 30 | | |
|  |  | 3400933898434 | LAMICTAL 25MG CPR DISP 30 | | |
|  |  | 3400933898663 | LAMICTAL 100MG CPR DISP +S 30 | | |
|  |  | 3400933898663 | LAMICTAL 100MG CPR DISP 30 | | |
|  |  | 3400934147135 | LAMICTAL 50MG CPR DISP 30 | | |
|  |  | 3400934147135 | LAMICTAL 50MG CPR DISP SEC ENF 30 | | |
|  |  | 3400934147364 | LAMICTAL 200MG CPR DISP 30 | | |
|  |  | 3400934147364 | LAMICTAL 200MG CPR DISP SE 30 | | |
|  |  | 3400934483561 | LAMICTAL 5MG CPR DISP 30 | | |
|  |  | 3400935458179 | LAMICTAL 2MG CPR DISP 30 | | |
|  | Generic | 3400921965919 | LAMOTRIGINE ARL 25MG CPR DISP 30 | | |
|  |  | 3400921965919 | LAMOTRIGINE ARW 25MG CPR DISP 30 | | |
|  |  | 3400921966688 | LAMOTRIGINE ARL 50MG CPR DISP 30 | | |
|  |  | 3400921966688 | LAMOTRIGINE ARW 50MG CPR DISP 30 | | |
|  |  | 3400921967289 | LAMOTRIGINE ARL 100MG CPR DISP 30 | | |
|  |  | 3400921967289 | LAMOTRIGINE ARW 100MG CPR DISP 30 | | |
|  |  | 3400921967920 | LAMOTRIGINE ARL 200MG CPR DISP 30 | | |
|  |  | 3400921967920 | LAMOTRIGINE ARW 200MG CPR DISP 30 | | |
|  |  | 3400936967175 | LAMOTRIGINE SDZ 25MG CPR DISP 30 | | |
|  |  | 3400936967816 | LAMOTRIGINE SDZ 100MG CPR DISP 30 | | |
|  |  | 3400936968417 | LAMOTRIGINE SDZ 200MG CPR DISP 30 | | |
|  |  | 3400937278898 | LAMOTRIGINE SDZ 50MG CPR DISP 30 | | |
|  |  | 3400937626217 | LAMOTRIGINE BGA 25MG CPR DISP 30 | | |
|  |  | 3400937627276 | LAMOTRIGINE BGA 50MG CPR DISP 30 | | |
|  |  | 3400937627795 | LAMOTRIGINE BGA 100MG CPR DISP 30 | | |
|  |  | 3400937628228 | LAMOTRIGINE BGA 200MG CPR DISP 30 | | |
|  |  | 3400937867979 | LAMOTRIGINE EG 200MG CPR DISP 30 | | |
|  |  | 3400937870290 | LAMOTRIGINE EG 100MG CPR DISP 30 | | |
|  |  | 3400937871761 | LAMOTRIGINE EG 25MG CPR DISP 30 | | |
|  |  | 3400937911436 | LAMOTRIGINE ARW 25MG CPR DISP 30 | | |
|  |  | 3400937912266 | LAMOTRIGINE ARW 100MG CPR DISP 30 | | |
|  |  | 3400937912846 | LAMOTRIGINE ARW 200MG CPR DISP 30 | | |
|  |  | 3400937913386 | LAMOTRIGINE ARW 50MG CPR DISP 30 | | |
|  |  | 3400938080339 | LAMOTRIGINE VIATRIS 25MG CPR DISP 30 | | |
|  |  | 3400938083989 | LAMOTRIGINE VIATRIS 50MG CPR DISP 30 | | |
|  |  | 3400938084931 | LAMOTRIGINE VIATRIS 100MG CPR DISP 30 | | |
|  |  | 3400938086072 | LAMOTRIGINE VIATRIS 200MG CPR DISP 30 | | |
|  |  | 3400939281742 | LAMOTRIGINE TVC 5MG CPR DISP 30 | | |
|  |  | 3400939282114 | LAMOTRIGINE TVC 25MG CPR DISP 30 | | |
|  |  | 3400939282572 | LAMOTRIGINE TVC 50MG CPR DISP 30 | | |
|  |  | 3400939283005 | LAMOTRIGINE TVC 100MG CPR DISP 30 | | |
|  |  | 3400939283524 | LAMOTRIGINE TVC 200MG CPR DISP 30 | | |
| OXC | Princeps | 3400935357014 | TRILEPTAL 150MG CPR 50 | |  |
|  |  | 3400935357243 | TRILEPTAL 300MG CPR 50 | |  |
|  |  | 3400935357472 | TRILEPTAL 600MG CPR 50 | |  |
|  |  | 3400935790125 | TRILEPTAL 60MG/ML SUSP BUV FL 250ML 1 | | |
|  |  | 3400949000326 | TRILEPTAL 600MG CPR (AIP) 50 | | |
|  | Generic | 3400922366005 | OXCARBAZEPINE SDZ 150MG CPR 50 | | |
|  |  | 3400922368016 | OXCARBAZEPINE SDZ 300MG CPR 50 | | |
|  |  | 3400922368993 | OXCARBAZEPINE SDZ 600MG CPR 50 | | |
|  |  | 3400930259974 | OXCARBAZEPINE VIATRIS 300MG CPR 50 | | |
|  |  | 3400937978781 | OXCARBAZEPINE MYL 150MG CPR 50 | | |
|  |  | 3400937978781 | OXCARBAZEPINE VTS150MG CPR 50 | | |
|  |  | 3400937982863 | OXCARBAZEPINE MYL 300MG CPR 50 | | |
|  |  | 3400937983464 | OXCARBAZEPINE MYL 600MG CPR 50 | | |
|  |  | 3400937983464 | OXCARBAZEPINE VIATRIS 600MG CPR 50 | | |
|  |  | 3400939675763 | OXCARBAZEPINE TVC 150MG CPR 50 | | |
|  |  | 3400939867403 | OXCARBAZEPINE TVC 600MG CPR 50 | | |
|  |  | 3400939867571 | OXCARBAZEPINE TVC 300MG CPR 50 | | |
| PB | Princeps | 3400930022290 | ALEPSAL 50MG CPR 30 | |  |
|  |  | 3400930425077 | GARDENAL 10MG CPR 80 | |  |
|  |  | 3400930425138 | GARDENAL 50MG CPR 30 | |  |
|  |  | 3400930425367 | GARDENAL 100MG CPR 20 | | |
|  |  | 3400930554968 | KANEURON 5,4% SOL BUV GTTE 1/30 ML | | |
|  |  | 3400932896103 | ALEPSAL 100MG CPR 30 | |  |
|  |  | 3400932896271 | ALEPSAL 15MG CPR 30 | |  |
|  |  | 3400933133085 | ALEPSAL 150MG CPR 30 | |  |
| PER | Princeps | 3400926776008 | FYCOMPA 2MG CPR 7 | |  |
|  |  | 3400926776237 | FYCOMPA 4MG CPR 28 | |  |
|  |  | 3400926776527 | FYCOMPA 6MG CPR 28 | |  |
|  |  | 3400926776756 | FYCOMPA 8MG CPR 28 | |  |
|  |  | 3400926776985 | FYCOMPA 10MG CPR 28 | |  |
|  |  | 3400926777128 | FYCOMPA 12MG CPR 28 | |  |
|  |  | 3400930174234 | FYCOMPA 0,5MG/ML SUSP BUV 1 | | |
| PGB | Princeps | 3400930183538 | LYRICA 20MG/ML SOL BUV 1 | | |
|  |  | 3400936512702 | LYRICA 25MG GELULE 56 | |  |
|  |  | 3400936512870 | LYRICA 50MG GELULE 84 | |  |
|  |  | 3400936512931 | LYRICA 75MG GELULE 56 | |  |
|  |  | 3400936513013 | LYRICA 100MG GELULE 84 | | |
|  |  | 3400936513181 | LYRICA 150MG GELULE 56 | | |
|  |  | 3400936513242 | LYRICA 200MG GELULE 84 | | |
|  |  | 3400936513303 | LYRICA 300MG GELULE 56 | | |
|  |  | 3400936513532 | LYRICA 25MG GELULE 84 | |  |
|  |  | 3400941701474 | LYRICA 20MG/ML SOL BUV 1 | | |
|  | Generic | 3400927938030 | PREGABALINE PFZ 25MG GELULE 56 | | |
|  |  | 3400927938269 | PREGABALINE PFZ 25MG GELULE 84 | | |
|  |  | 3400927938320 | PREGABALINE PFZ 50MG GELULE 84 | | |
|  |  | 3400927938498 | PREGABALINE PFZ 75MG GELULE 56 | | |
|  |  | 3400927938559 | PREGABALINE PFZ 100MG GELULE 84 | | |
|  |  | 3400927938610 | PREGABALINE PFZ 150MG GELULE 56 | | |
|  |  | 3400927938849 | PREGABALINE PFZ 300MG GELULE 56 | | |
|  |  | 3400930018118 | PREGABALINE MYL 25MG GELULE 56 | | |
|  |  | 3400930018125 | PREGABALINE MYL 25MG GELULE 84 | | |
|  |  | 3400930018149 | PREGABALINE MYL 50MG GELULE 84 | | |
|  |  | 3400930018163 | PREGABALINE MYL 75MG GELULE 56 | | |
|  |  | 3400930018187 | PREGABALINE MYL 100MG GELULE 84 | | |
|  |  | 3400930018200 | PREGABALINE MYL 150MG GELULE 56 | | |
|  |  | 3400930018224 | PREGABALINE MYL 200MG GELULE 84 | | |
|  |  | 3400930018248 | PREGABALINE MYL 300MG GELULE 56 | | |
|  |  | 3400930019252 | PREGABALINE SDZ GMBH 25MG GELULE 56 | | |
|  |  | 3400930019269 | PREGABALINE SDZ GMBH 25MG GELULE 84 | | |
|  |  | 3400930019276 | PREGABALINE SDZ GMBH 50MG GELULE 84 | | |
|  |  | 3400930019283 | PREGABALINE SDZ GMBH 75MG GELULE 56 | | |
|  |  | 3400930019290 | PREGABALINE SDZ GMBH 100MG GELULE 84 | | |
|  |  | 3400930019306 | PREGABALINE SDZ GMBH 150MG GELULE 56 | | |
|  |  | 3400930019313 | PREGABALINE SDZ GMBH 200MG GELULE 84 | | |
|  |  | 3400930019320 | PREGABALINE SDZ GMBH 300MG GELULE 56 | | |
|  |  | 3400930022627 | PREGABALINE TVS 25MG GELULE 56 | | |
|  |  | 3400930022634 | PREGABALINE TVS 25MG GELULE 84 | | |
|  |  | 3400930022689 | PREGABALINE TVS 50MG GELULE 84 | | |
|  |  | 3400930022733 | PREGABALINE TVS 75MG GELULE 56 | | |
|  |  | 3400930022771 | PREGABALINE TVS 100MG GELULE 84 | | |
|  |  | 3400930022818 | PREGABALINE TVS 150MG GELULE 56 | | |
|  |  | 3400930022849 | PREGABALINE TVS 200MG GELULE 84 | | |
|  |  | 3400930022863 | PREGABALINE TVS 300MG GELULE 56 | | |
|  |  | 3400930023778 | PREGABALINE ZEN 25MG GELULE 56 | | |
|  |  | 3400930023778 | PREGABALINE ZTL 25MG GELULE 56 | | |
|  |  | 3400930023785 | PREGABALINE ZEN 25MG GELULE 84 | | |
|  |  | 3400930023785 | PREGABALINE ZTL 25MG GELULE 84 | | |
|  |  | 3400930023792 | PREGABALINE ZEN 50MG GELULE 84 | | |
|  |  | 3400930023792 | PREGABALINE ZTL 50MG GELULE 84 | | |
|  |  | 3400930023808 | PREGABALINE ZEN 75MG GELULE 56 | | |
|  |  | 3400930023808 | PREGABALINE ZTL 75MG GELULE 56 | | |
|  |  | 3400930023815 | PREGABALINE ZEN 100MG GELULE 84 | | |
|  |  | 3400930023815 | PREGABALINE ZTL 100MG GELULE 84 | | |
|  |  | 3400930023839 | PREGABALINE ZEN 150MG GELULE 56 | | |
|  |  | 3400930023839 | PREGABALINE ZTL 150MG GELULE 56 | | |
|  |  | 3400930023846 | PREGABALINE ZEN 200MG GELULE 84 | | |
|  |  | 3400930023846 | PREGABALINE ZTL 200MG GELULE 84 | | |
|  |  | 3400930023853 | PREGABALINE ZEN 300MG GELULE 56 | | |
|  |  | 3400930023853 | PREGABALINE ZTL 300MG GELULE 56 | | |
|  |  | 3400930028902 | PREGABALINE EG 25MG GELULE 56 | | |
|  |  | 3400930028919 | PREGABALINE EG 25MG GELULE 84 | | |
|  |  | 3400930028940 | PREGABALINE EG 50MG GELULE 84 | | |
|  |  | 3400930028971 | PREGABALINE EG 75MG GELULE 56 | | |
|  |  | 3400930028995 | PREGABALINE EG 100MG GELULE 84 | | |
|  |  | 3400930029015 | PREGABALINE EG 150MG GELULE 56 | | |
|  |  | 3400930029077 | PREGABALINE EG 300MG GELULE 56 | | |
|  |  | 3400930031896 | PREGABALINE EVO 25MG GELULE 56 | | |
|  |  | 3400930031971 | PREGABALINE EVO 50MG GELULE 84 | | |
|  |  | 3400930032190 | PREGABALINE EVO 75MG GELULE 56 | | |
|  |  | 3400930032534 | PREGABALINE EVO 300MG GELULE 56 | | |
|  |  | 3400930032664 | PREGABALINE BGA 25MG GELULE 56 | | |
|  |  | 3400930032671 | PREGABALINE BGA 25MG GELULE 84 | | |
|  |  | 3400930032749 | PREGABALINE BGA 50MG GELULE 84 | | |
|  |  | 3400930032916 | PREGABALINE BGA 75MG GELULE 56 | | |
|  |  | 3400930032954 | PREGABALINE BGA 100MG GELULE 84 | | |
|  |  | 3400930033074 | PREGABALINE BGA 150MG GELULE 56 | | |
|  |  | 3400930033111 | PREGABALINE BGA 200MG GELULE 84 | | |
|  |  | 3400930033234 | PREGABALINE BGA 300MG GELULE 56 | | |
|  |  | 3400930040249 | PREGABALINE ACC 300MG GELULE 56 | | |
|  |  | 3400930040294 | PREGABALINE ARW 25MG GELULE 56 | | |
|  |  | 3400930040300 | PREGABALINE ARW 25MG GELULE 84 | | |
|  |  | 3400930040386 | PREGABALINE ARW 50MG GELULE 84 | | |
|  |  | 3400930040416 | PREGABALINE ARW 75MG GELULE 56 | | |
|  |  | 3400930040461 | PREGABALINE ARW 100MG GELULE 84 | | |
|  |  | 3400930040492 | PREGABALINE ARW 150MG GELULE 56 | | |
|  |  | 3400930040546 | PREGABALINE ARW 200MG GELULE 84 | | |
|  |  | 3400930040584 | PREGABALINE ARW 300MG GELULE 56 | | |
|  |  | 3400930041352 | PREGABALINE HCS 25MG GELULE 56 | | |
|  |  | 3400930041406 | PREGABALINE HCS 50MG GELULE 84 | | |
|  |  | 3400930041420 | PREGABALINE HCS 75MG GELULE 56 | | |
|  |  | 3400930056882 | PREGABALINE CRT 25MG GELULE 56 | | |
|  |  | 3400930056943 | PREGABALINE CRT 50MG GELULE 84 | | |
|  |  | 3400930060308 | PREGABALINE CRT 75MG GELULE 56 | | |
|  |  | 3400930060391 | PREGABALINE CRT 300MG GELULE 56 | | |
|  |  | 3400930085141 | PREGABALINE ZTK 25MG GELULE 56 | | |
|  |  | 3400930085158 | PREGABALINE ZTK 25MG GELULE 84 | | |
|  |  | 3400930085172 | PREGABALINE ZTK 50MG GELULE 84 | | |
|  |  | 3400930085189 | PREGABALINE ZTK 75MG GELULE 56 | | |
|  |  | 3400930085196 | PREGABALINE ZTK 100MG GELULE 84 | | |
|  |  | 3400930085202 | PREGABALINE ZTK 150MG GELULE 56 | | |
|  |  | 3400930085219 | PREGABALINE ZTK 200MG GELULE 84 | | |
|  |  | 3400930085233 | PREGABALINE ZTK 300MG GELULE 56 | | |
|  |  | 3400930087183 | PREGABALINE SDZ 25MG GELULE 56 | | |
|  |  | 3400930087190 | PREGABALINE SDZ 25MG GELULE 84 | | |
|  |  | 3400930087206 | PREGABALINE SDZ 50MG GELULE 84 | | |
|  |  | 3400930087213 | PREGABALINE SDZ 75MG GELULE 56 | | |
|  |  | 3400930087220 | PREGABALINE SDZ 100MG GELULE 84 | | |
|  |  | 3400930087244 | PREGABALINE SDZ 200MG GELULE 84 | | |
|  |  | 3400930087251 | PREGABALINE SDZ 300MG GELULE 56 | | |
|  |  | 3400930087268 | PREGABALINE SDZ 150MG GELULE 56 | | |
|  |  | 3400930089965 | PREGABALINE ZYD 75MG GELULE 56 | | |
|  |  | 3400930089989 | PREGABALINE ZYD 300MG GELULE 56 | | |
|  |  | 3400930128534 | PREGABALINE EGL 25MG GELULE 56 | | |
|  |  | 3400930128572 | PREGABALINE EGL 25MG GELULE 84 | | |
|  |  | 3400930128657 | PREGABALINE EGL 50MG GELULE 84 | | |
|  |  | 3400930128695 | PREGABALINE EGL 75MG GELULE 56 | | |
|  |  | 3400930128787 | PREGABALINE EGL 100MG GELULE 84 | | |
|  |  | 3400930128848 | PREGABALINE EGL 150MG GELULE 56 | | |
|  |  | 3400930128923 | PREGABALINE EGL 200MG GELULE 84 | | |
|  |  | 3400930129159 | PREGABALINE EGL 300MG GELULE 56 | | |
|  |  | 3400930131596 | PREGABALINE ZYD 25MG GELULE 56 | | |
|  |  | 3400930147689 | PREGABALINE ARW 25MG GELULE 56 | | |
|  |  | 3400930147696 | PREGABALINE ARW 25MG GELULE 84 | | |
|  |  | 3400930147757 | PREGABALINE ARW 50MG GELULE 84 | | |
|  |  | 3400930147801 | PREGABALINE ARW 75MG GELULE 56 | | |
|  |  | 3400930147856 | PREGABALINE ARW 100MG GELULE 84 | | |
|  |  | 3400930147894 | PREGABALINE ARW 150MG GELULE 56 | | |
|  |  | 3400930147955 | PREGABALINE ARW 200MG GELULE 84 | | |
|  |  | 3400930147979 | PREGABALINE ARW 300MG GELULE 56 | | |
|  |  | 3400930195260 | PREGABALINE CRT 100MG GELULE 84 | | |
|  |  | 3400930238479 | PREGABALINE ZTL 25MG 1 BOITE DE 56, GELULE S | | |
|  |  | 3400930238486 | PREGABALINE ZTL 25MG 1 BOITE DE 84, GELULE S | | |
| PHT | Princeps | 3400930303030 | DI HYDAN 100MG CPR 60 | |  |
| PRM | Princeps | 3400930698259 | MYSOLINE 250MG CPR 50 | |  |
| RFM | Princeps | 3400922069128 | INOVELON 40MG/ML SUSP BUVABLE 1 | | |
|  |  | 3400938176124 | INOVELON 200MG CPR 60 | |  |
|  |  | 3400938176292 | INOVELON 400MG CPR 60 | |  |
|  |  | 3400938284614 | INOVELON 100MG CPR 10 | |  |
| STP | Princeps | 3400937832212 | DIACOMIT 250MG GELULE 60 | | |
|  |  | 3400937832502 | DIACOMIT 500MG GELULE 60 | | |
|  |  | 3400937832960 | DIACOMIT 250MG PDR PR SUSP BUV 60 | | |
|  |  | 3400937833271 | DIACOMIT 500MG PDR PR SUSP BUV 60 | | |
| TPM | Princeps | 3400934830518 | EPITOMAX 15MG GELULE 28 | | |
|  |  | 3400934830686 | EPITOMAX 25MG GELULE 28 | | |
|  |  | 3400934830747 | EPITOMAX 50MG GELULE 28 | | |
|  |  | 3400935634139 | EPITOMAX 50MG CPR 28 | |  |
|  |  | 3400935634429 | EPITOMAX 100MG CPR 28 | |  |
|  |  | 3400935634658 | EPITOMAX 200MG CPR 28 | |  |
|  | Generic | 3400930194850 | TOPIRAMATE ARL 100MG CPR 30 | | |
|  |  | 3400930194850 | TOPIRAMATE ARW 100MG CPR 30 | | |
|  |  | 3400930194867 | TOPIRAMATE ARL 50MG CPR 30 | | |
|  |  | 3400930194867 | TOPIRAMATE ARW 50MG CPR 30 | | |
|  |  | 3400938052183 | TOPIRAMATE BGA 50MG CPR 28 | | |
|  |  | 3400938056327 | TOPIRAMATE BGA 100MG CPR 28 | | |
|  |  | 3400938057386 | TOPIRAMATE BGA 200MG CPR 28 | | |
|  |  | 3400938058109 | TOPIRAMATE EG 50MG CPR 28 | | |
|  |  | 3400938059397 | TOPIRAMATE EG 100MG CPR 28 | | |
|  |  | 3400938270426 | TOPIRAMATE MYL 50MG CPR 28 | | |
|  |  | 3400938270426 | TOPIRAMATE VIATRIS 50MG CPR 28 | | |
|  |  | 3400938270884 | TOPIRAMATE MYL 100MG CPR 28 | | |
|  |  | 3400938270884 | TOPIRAMATE VIATRIS 100MG CPR 28 | | |
|  |  | 3400938271256 | TOPIRAMATE MYL 200MG CPR 28 | | |
|  |  | 3400938465075 | TOPIRAMATE ARW 50MG CPR 28 | | |
|  |  | 3400938467086 | TOPIRAMATE ARW 100MG CPR 28 | | |
|  |  | 3400938471229 | TOPIRAMATE ARW 50MG GELULE 28 | | |
|  |  | 3400938679755 | TOPIRAMATE TVC 50MG CPR 30 | | |
|  |  | 3400938680706 | TOPIRAMATE TVC 100MG CPR 30 | | |
|  |  | 3400938979305 | TOPIRAMATE ARG 100MG CPR 30 | | |
|  |  | 3400939000695 | TOPIRAMATE ARG 50MG CPR 30 | | |
|  |  | 3400939016955 | TOPIRAMATE SDZ 100MG CPR 28 | | |
|  |  | 3400939138015 | TOPIRAMATE BGR 50MG CPR 28 | | |
|  |  | 3400939138473 | TOPIRAMATE BGR 100MG CPR 28 | | |
|  |  | 3400939138473 | TOPIRAMATE ZYD 100MG CPR 28 | | |
|  |  | 3400939139135 | TOPIRAMATE BGR 200MG CPR 28 | | |
|  |  | 3400939171302 | TOPIRAMATE ZYD 25MG GELULE 28 | | |
|  |  | 3400939434698 | TOPIRAMATE SDZ 50MG CPR 28 | | |
|  |  | 3400939863900 | TOPIRAMATE ARW 25MG GELULE 28 | | |
| VGB | Princeps | 3400930157602 | KIGABEQ 100MG CPR 100 | |  |
|  |  | 3400930157626 | KIGABEQ 500MG CPR 50 | |  |
|  |  | 3400933780418 | SABRIL 500MG CPR 60 | |  |
|  |  | 3400933780647 | SABRIL 500MG GRANULES 60 | | |
| VPA | Princeps | 3400930212547 | DIVALCOTE 250MG CPR GASTRO R 30 | | |
|  |  | 3400930212578 | DIVALCOTE 500MG CPR GASTRO R 90 | | |
|  |  | 3400930292921 | DEPAKINE 200MG CPR 40 | |  |
|  |  | 3400930293003 | DEPAKINE 200MG/ML SOL BUV FL 40ML 1 | | |
|  |  | 3400931922766 | DEPAKINE 500MG CPR 40 | |  |
|  |  | 3400932634507 | DEPAKINE 57,64MG/ML SIROP FL 150ML 1 | | |
|  |  | 3400933018023 | DEPAKINE CHRONO 500MG CPR 30 | | |
|  |  | 3400934876233 | DEPAKOTE 250MG CPR 30 | | |
|  |  | 3400935372777 | DEPAKINE CHRONO 500MG, CPR 100 | | |
|  |  | 3400935444271 | DEPAKOTE 500MG CPR 90 | | |
|  |  | 3400936551152 | MICROPAKINE LP 100MG GRANULE SAC 30 | | |
|  |  | 3400936551152 | MICROPAKINE LP 100MG GRANULE SACHET 30 | | |
|  |  | 3400936551213 | MICROPAKINE LP 250MG GRANULE SAC 30 | | |
|  |  | 3400936551213 | MICROPAKINE LP 250MG GRANULE SACHET 30 | | |
|  |  | 3400936551381 | MICROPAKINE LP 500MG GRANULE SAC 30 | | |
|  |  | 3400936551381 | MICROPAKINE LP 500MG GRANULE SACHET 30 | | |
|  |  | 3400936551442 | MICROPAKINE LP 750MG GRANULE SAC 30 | | |
|  |  | 3400936551442 | MICROPAKINE LP 750MG GRANULE SACHET 30 | | |
|  |  | 3400936551732 | MICROPAKINE LP 1000MG GRANULE SAC 30 | | |
|  |  | 3400936551732 | MICROPAKINE LP 1000MG GRANULE SACHET 30 | | |
|  | Generic | 3400936738577 | VALPROATE NA ZTL 200MG CPR 40 | | |
|  |  | 3400936738638 | VALPROATE NA ZTL 500MG CPR 40 | | |
|  |  | 3400936738867 | VALPROATE NA ZTL 20 P. 100 FL 1/40 ML SIR | | |
|  |  | 3400936934696 | VALPROATE NA ZTL LP 500MG CPR 30 | | |
|  |  | 3400936965973 | VALPROATE NA EG LP 500MG CPR 30 | | |
|  |  | 3400936966116 | VALPROATE NA TVS LP 500MG CPR 30 | | |
|  |  | 3400936966284 | VALPROATE NA TVS LP 500MG CPR 30 | | |
|  |  | 3400936966406 | VALPROATE NA TVC LP 500MG CPR 30 | | |
|  |  | 3400936966574 | VALPROATE NA RPG LP 500MG CPR 30 | | |
|  |  | 3400936966925 | VALPROATE NA BGA LP 500MG CPR 30 | | |
|  |  | 3400936967007 | VALPROATE NA MYL LP 500MG CPR 30 | | |
|  |  | 3400936967007 | VALPROATE NA VIATRIS LP 500MG CPR 30 | | |
|  |  | 3400937157452 | VALPROATE NA ARW LP 500MG CPR 30 | | |
|  |  | 3400939848532 | VALPROATE NA SDZ 500MG CPR 30 | | |
|  |  | 3400949980246 | VALPROATE NA ARW 200MG/ML BUV 40ML 1 | | |
| ZNS | Princeps | 3400936594265 | ZONEGRAN 25MG GELULE 14 | | |
|  |  | 3400936594555 | ZONEGRAN 100MG GELULE 56 | | |
|  |  | 3400936920330 | ZONEGRAN 50MG GELULE 14 | | |
|  | Generic | 3400930058497 | ZONISAMIDE SDZ 25MG GELULE 14 | | |
|  |  | 3400930058510 | ZONISAMIDE SDZ 50MG GELULE 14 | | |
|  |  | 3400930058589 | ZONISAMIDE SDZ 100MG GELULE 56 | | |
|  |  | 3400930058794 | ZONISAMIDE MYL 25MG GELULE 14 | | |
|  |  | 3400930058824 | ZONISAMIDE MYL 50MG GELULE 14 | | |
|  |  | 3400930058886 | ZONISAMIDE MYL 100MG GELULE 56 | | |
|  |  | 3400930100776 | ZONISAMIDE TVC 25MG GELULE 14 | | |
|  |  | 3400930100813 | ZONISAMIDE TVC 50MG GELULE 14 | | |
|  |  | 3400930100868 | ZONISAMIDE TVC 100MG GELULE 56 | | |
|  |  | 3400930211140 | ZONISAMIDE NEURAXPHARM 25MG CPR 14 | | |

# Supplementary Table 3. Annual population aged < 20 years in France, 2014–2024

Over the period of this study, the French population aged < 20 years remained almost stable, showing a slight decrease from 15.6 million in 2014 to 15.3 million in 2023 (-2 %) according to national demographic data. When expressed relative to population size, the prevalence of treated children therefore increased from approximately 13 to 16 per 1000, corresponding to a ~25-28 % relative rise. Note that population figures cover individuals aged < 20 years, whereas ASM prescribing data refer to individuals aged < 19 years, which may introduce a minor denominator mismatch.

| Year | Population aged < 20 years (n) | Annual change (%) |
| --- | --- | --- |
| 2014 | 15 588 708 | - |
| 2015 | 15 651 778 | +0.4 |
| 2016 | 15 645 526 | -0.04 |
| 2017 | 15 616 148 | -0.2 |
| 2018 | 15 611 515 | -0.03 |
| 2019 | 15 584 916 | -0.2 |
| 2020 | 15 512 858 | -0.5 |
| 2021 | 15 417 227 | -0.6 |
| 2022 | 15 347 319 | -0.5 |
| 2023 | 15 257 551 | -0.6 |

Data derived from the Institut national d’études démographiques (INED), Population by age, France, [https://www.ined.fr/en/everything_about_population/data/france/population-structure/population-age/](https://www.ined.fr/en/everything_about_population/data/france/population-structure/population-age/?utm_source=chatgpt.com)

# Supplementary Table 4. Odds ratios for female vs male prescriptions by ASM, prescriber type, and calendar year

BRV: brivaracetam; CBZ: carbamazepine; CBD: cannabidiol; CNB: cenobamate; ESL: eslicarbazepine; ESM: ethosuximide; EVER: everolimus; FFA: fenfluramine; GBP: gabapentin; LCM: lacosamide; LEV: levetiracetam; LTG: lamotrigine; OXC: oxcarbazepine; PB: phenobarbital; PER: perampanel; PGB: pregabalin; PHT: phenytoin; PRM: primidone; RFM: rufinamide; STP: stiripentol; TPM: topiramate; VGB: vigabatrin; VPA: valproic acid; ZNS: zonisamide.

| Factor | Category | OR | 95%  Confidence Interval | Predominant sex |
| --- | --- | --- | --- | --- |
| Year (per 1 year) | Year | 0.992 | 0.991-0.993 | Males |
| BRV | ASM | 1.14 | 1.06-1.22 | Females |
| CBD | ASM | 0.87 | 0.78-0.97 | Males |
| CBZ | ASM | 0.76 | 0.74-0.78 | Males |
| CNB | ASM | 0.96 | 0.82-1.13 | No significant difference |
| ESL | ASM | 0.62 | 0.58-0.67 | Males |
| ESM | ASM | 1.22 | 1.19-1.26 | Females |
| EVER | ASM | 0.93 | 0.83-1.05 | No significant difference |
| FFA | ASM | 1.34 | 0.98-1.84 | No significant difference |
| GBP | ASM | 1.48 | 1.45-1.52 | Females |
| LEV | ASM | 1.12 | 1.09-1.14 | Females |
| LTG | ASM | 1.66 | 1.63-1.70 | Females |
| OXC | ASM | 0.82 | 0.80-0.84 | Males |
| PB | ASM | 0.78 | 0.74-0.82 | Males |
| PER | ASM | 1.08 | 1.04-1.11 | Females |
| PGB | ASM | 1.49 | 1.46-1.53 | Females |
| PHT | ASM | 0.86 | 0.77-0.95 | Males |
| PRM | ASM | 0.69 | 0.57-0.83 | Males |
| RFM | ASM | 0.85 | 0.81-0.89 | Males |
| STP | ASM | 0.90 | 0.86-0.95 | Males |
| TPM | ASM | 1.43 | 1.40-1.46 | Females |
| VGB | ASM | 0.87 | 0.85-0.90 | Males |
| VPA | ASM | 0.56 | 0.55-0.57 | Males |
| ZNS | ASM | 1.17 | 1.13-1.20 | Females |
| Hospital practitioner | Prescriber | 0.97 | 0.96-0.98 | Males |
| Other | Prescriber | 0.51 | 0.44-0.58 | Males |
| Private neurologist | Prescriber | 1.37 | 1.35-1.39 | Females |
| Private paediatrician | Prescriber | 1.04 | 1.02-1.06 | Females |
| Private psychiatrist | Prescriber | 2.67 | 2.58-2.76 | Females |
| Unknown | Prescriber | 1.01 | 1.00-1.03 | Females |

# ****Supplementary Table 5. Log-linear Gamma model for annual reimbursed expenditure on paediatric antiseizure medicines in France (excluding cannabidiol)****

Annual reimbursed expenditure is a continuous and over-dispersed outcome. Although a Poisson log-linear model was fitted initially to explore long-term trends after exclusion of cannabidiol, a Gamma regression with a log link was retained as the primary and more appropriate model. The Gamma model showed a small, non-significant annual increase of approximately 1% in total ASM expenditure (rate ratio = 1.01, 95% CI 1.00–1.01; p = 0.11), confirming the overall stability of spending between 2014 and 2023 once cannabidiol was excluded.

Gamma regression with a log link

| Parameter | Estimate (β̂) | Standard error | 95 % CI | p-value |
| --- | --- | --- | --- | --- |
| Intercept (2014 baseline) | 957 237 | 5.61 | 0.017 - 54 397 744 | 0.26 |
| Year | 1.005 | 0.0028 | 1.000 - 1.01 | 0.11 |

Supplementary Table 6. Factors associated with generic uptake of antiseizure medications
Odds ratios (ORs) with 95% bootstrap confidence intervals from a LASSO logistic regression model (500 replications). Zonisamide was the reference antiseizure medications (ASM) and general practitioners the reference prescriber. OR>1 indicates higher odds of generic prescription.

| Factor | OR | 95% CI | Side with higher odds |
| --- | --- | --- | --- |
| ASM: Gabapentin | 30.11 | 20.05-46.66 | Generic > |
| ASM: Pregabalin | 11.73 | 8.44-17.32 | Generic > |
| Prescriber: Private psychiatrists | 2.47 | 1.50-3.85 | Generic > |
| ASM: Lamotrigine | 1.80 | 1.29-2.56 | Generic > |
| ASM: Levetiracetam | 1.34 | 1.00-1.96 | Generic > |
| Year (per 1 unit) | 1.09 | 1.06-1.13 | Generic > |
| ASM: Zonisamide | 1.00 | 0.91-1.08 | Originator > |
| Prescriber: General practitioner | 1.00 | 1.00-1.22 | Originator > |
| Prescriber: Hospital practitioners | 1.00 | 1.00-1.21 | Originator > |
| Prescriber: Other | 1.00 | 0.65-1.94 | Originator > |
| ASM: Oxcarbazepine | 0.71 | 0.49-1.00 | Originator > |
| ASM: Carbamazepine | 0.48 | 0.32-0.72 | Originator > |
| ASM: Topiramate | 0.39 | 0.28-0.56 | Originator > |
| ASM: Valproic acid | 0.35 | 0.24-0.50 | Originator > |
| Prescriber: Private neurologist | 0.23 | 0.16-0.32 | Originator > |
| Prescriber: Private paediatrician | 0.04 | 0.03-0.06 | Originator > |

# Supplementary Figure 1: Timeline of Market Authorization for Epilepsy-Indicated Medicines by Pharmacological Generation in France

Antiseizure medicines can be conveniently grouped into three chronological “generations,” each reflecting distinct eras of discovery, pharmacology, and safety monitoring. Horizontal bars position each molecule at its year of first French marketing authorization. Colour coding distinguishes generations: dark blue = first generation (1909-1958); teal = second generation (1958-1989); orange = third generation (1989-2023). Third‑generation drugs were subdivided into early (1990-1999), mid (2000-2009) and late (2010-2023) subclasses. Molecule labels appear above each bar; overlapping labels are staggered for clarity. The figure illustrates the progressive acceleration of drug introductions and the predominance of third-generation agents over the past 30 years.

.


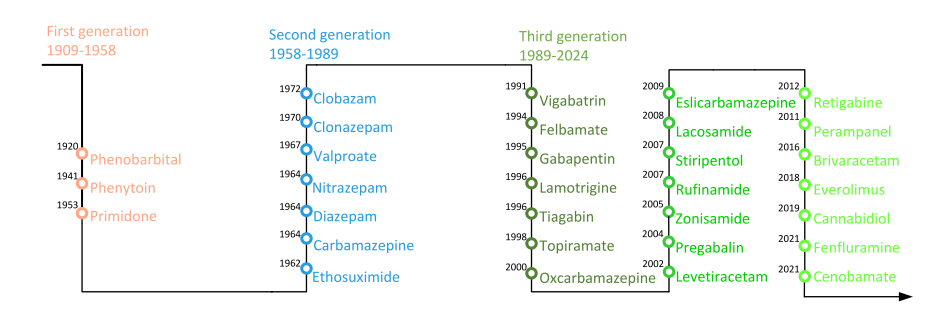


# Supplementary Figure 2: Flowchart of Data Selection for our Study


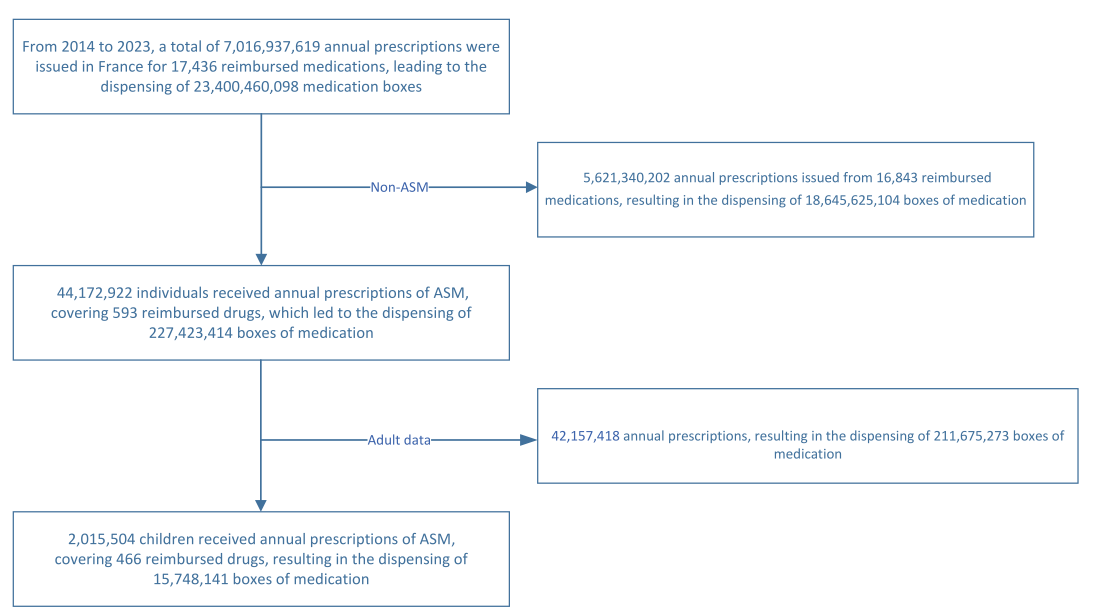


# Supplementary Figure 3: Evolution of treatment by ASM generations between 2014 and 2023


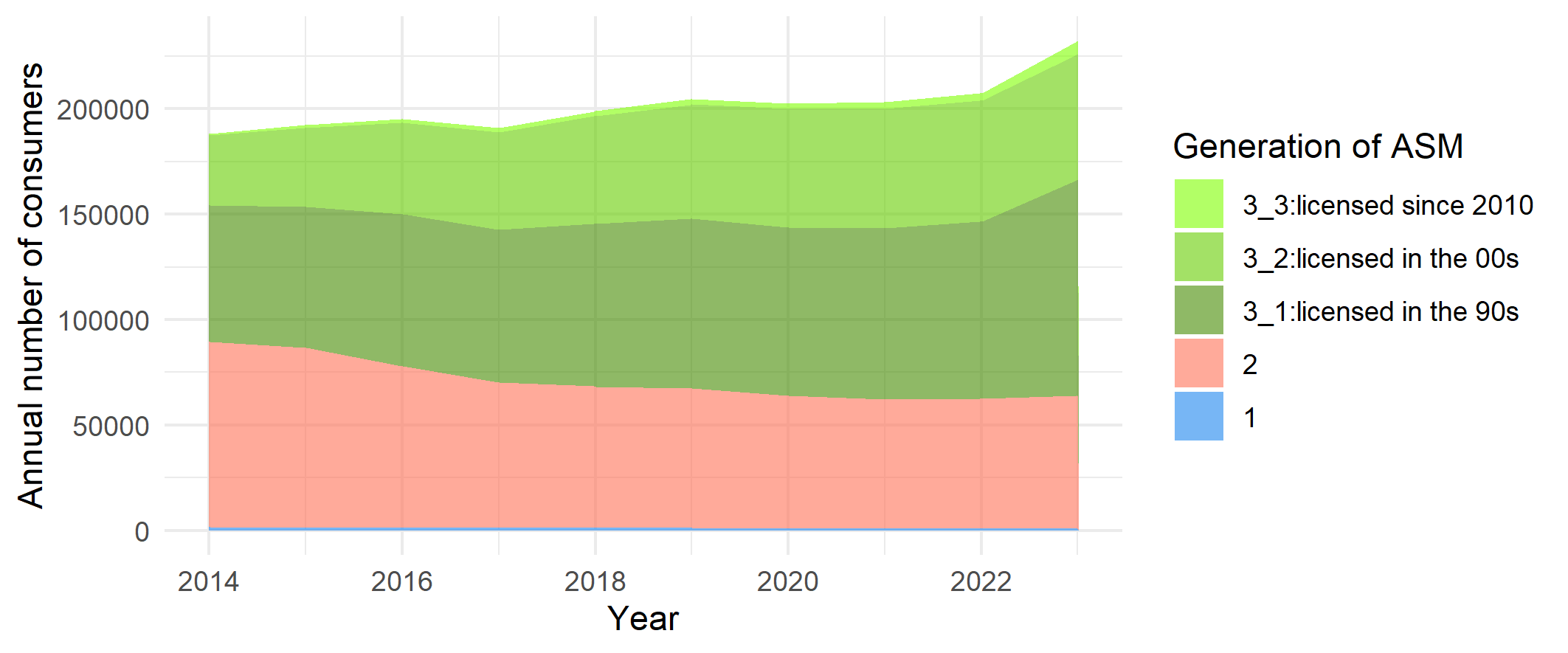


# Supplementary Figure 4: Total Annual Pediatrics Antiepileptic Drug Consumers Over the Past Decade

The main antiseizure medications prescribed to children over the past decade include brivaracetam (BRV), cannabidiol (CBD), carbamazepine (CBZ), cenobamate (CNB), eslicarbazepine (ESL), ethosuximide (ESM), everolimus (EVER), fenfluramine (FFA), gabapentin (GBP), levetiracetam (LEV), lacosamide (LCM), lamotrigine (LTG), oxcarbazepine (OXC), perampanel (PER), phenobarbital (PHB), pregabalin (PGB), phenytoin (PHT), primidone (PRM), rufinamide (RFM), stiripentol (STP), topiramate (TPM), vigabatrin (VGB), valproic acid (VPA), and zonisamide (ZNS)

# Supplementary Figure 5: Prescriber of paediatric Anti-Seizure Medications Over the Past Decade

# Supplementary Figure 6: Share of Prescriber Activity by Year


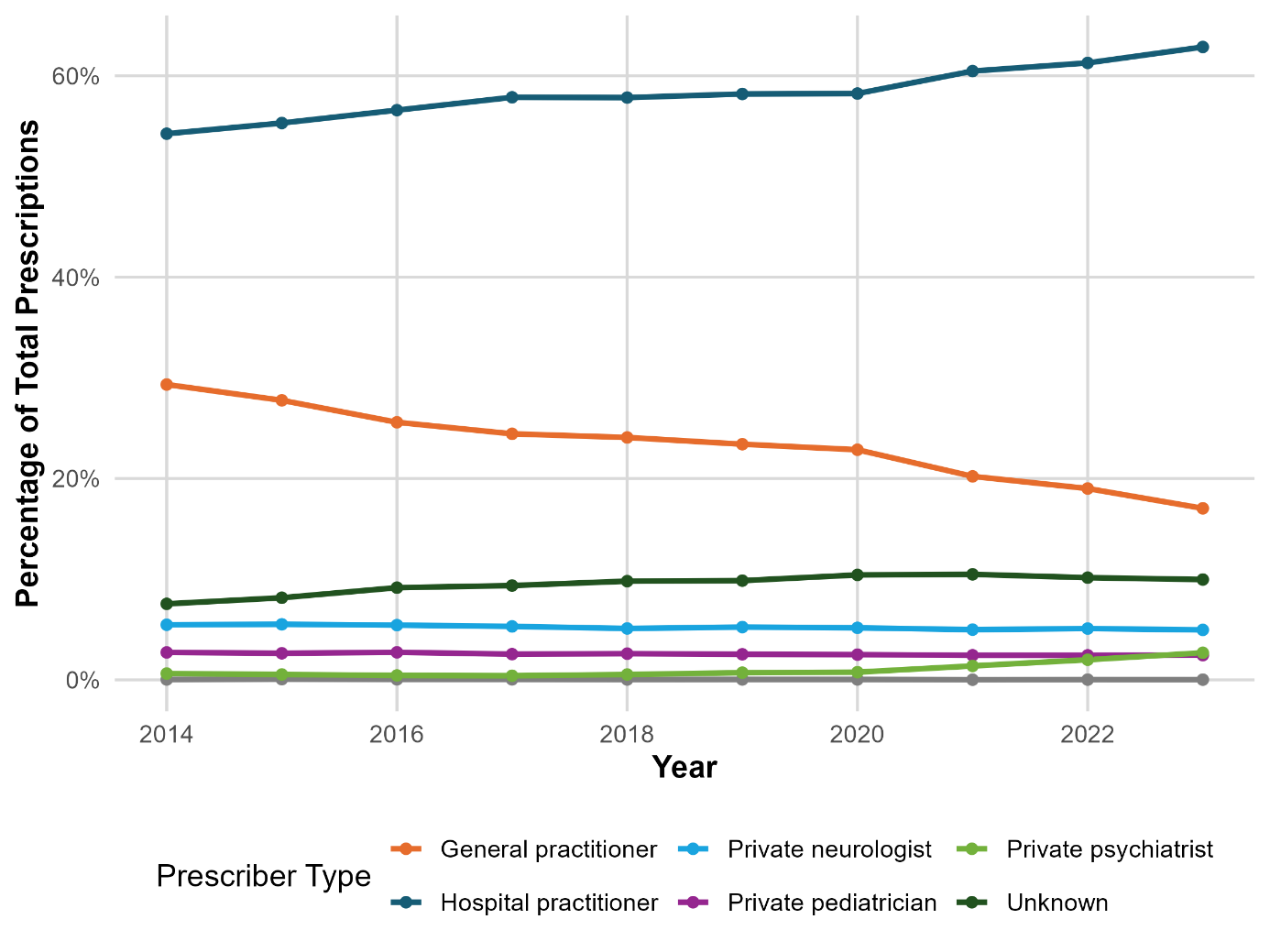


# Supplementary Figure 7: Leave-One-Year-Out Analysis Confirms Stable Association Between Valproate Exposure and Patient Sex

Leave-one-year-out logistic regression models show stable odds ratios (0.417-0.449) associating valproate exposure with female sex. Excluding 2017 slightly increases the OR, reflecting regulatory action that year, but does not alter the overall pattern.


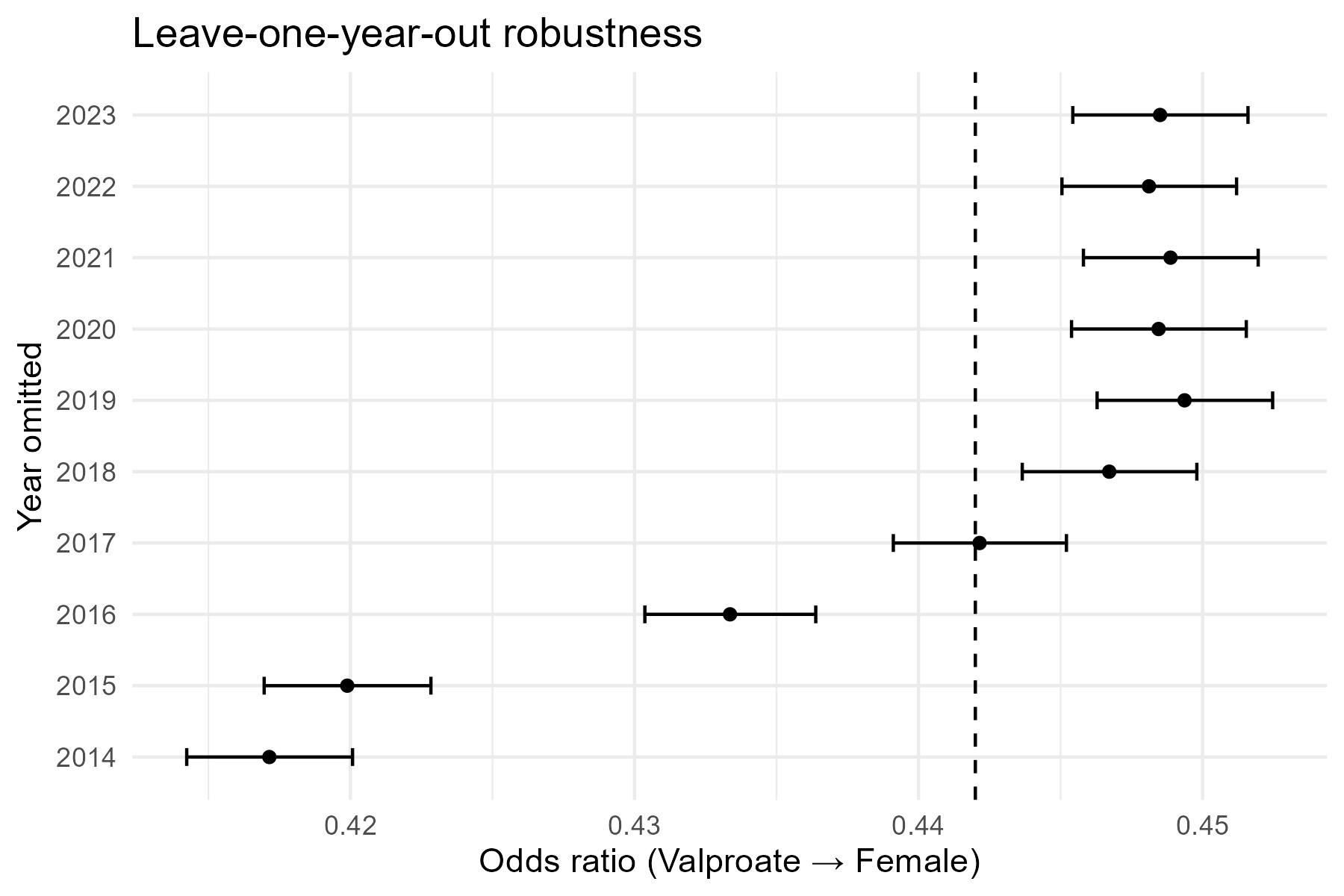


# Supplementary Figure 8: Annual reimbursed expenditure for paediatric antiseizure medicines in France, 2014-2023

Bars show total annual national spending on paediatric antiseizure medicines between 2014 and 2023, expressed in millions of euros. The reimbursed amount (REM) corresponds to the actual sum paid by the French National Health Insurance to pharmacies. Expenditure remained stable until 2022, followed by an ~80 % increase in 2023, mainly driven by the market entry of pharmaceutical-grade cannabidiol.


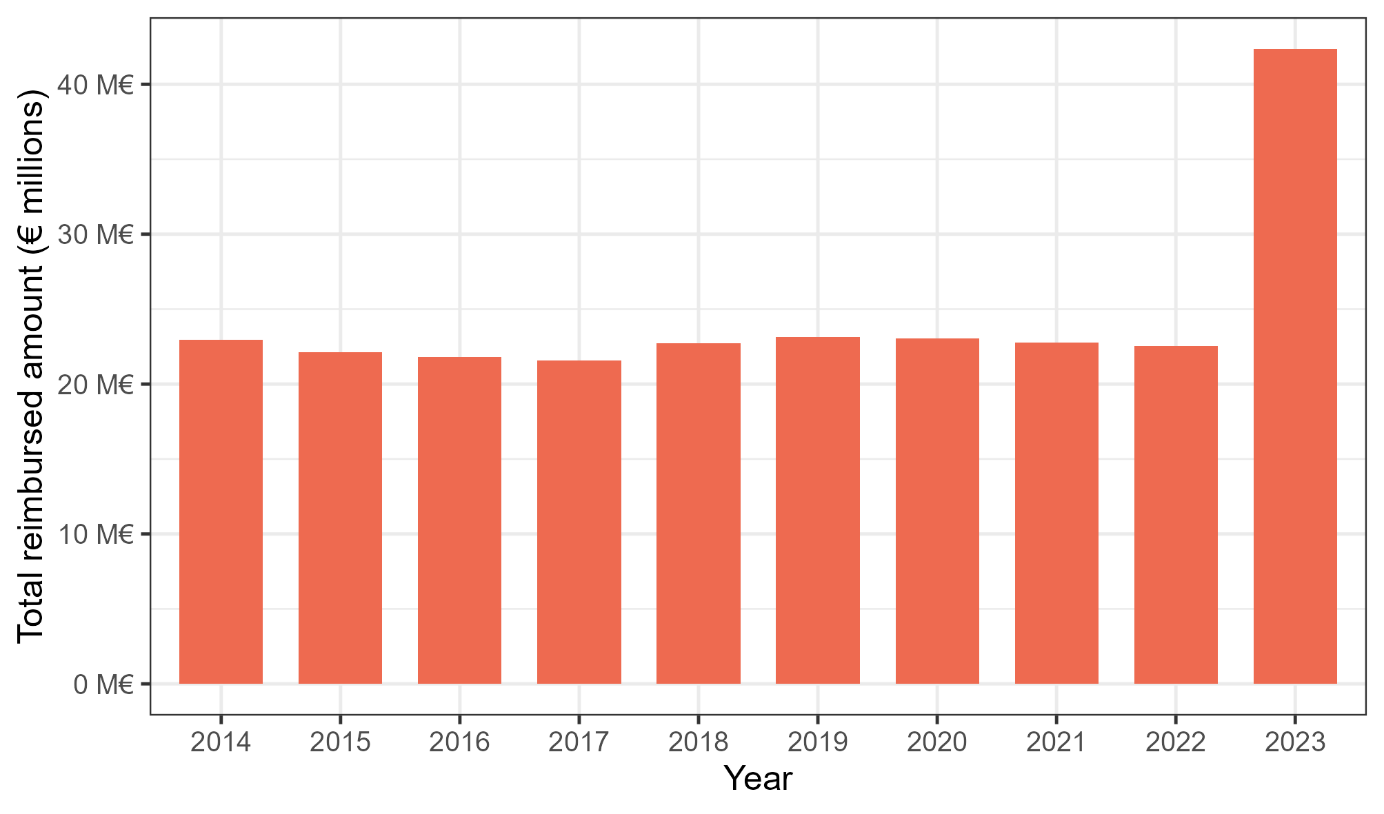


# Supplementary Figure 9: Annual per‑patient cost of podiatric anti‑seizure medicines dispensed in 2023

Panel A: Box plots shows the distribution of annual costs per patient across pharmacological generations. Costs increased significantly from first- to third-generation ASMs (Jonckheere-Terpstra test: p < 0·0001).

Panel B: Individual molecules, ordered by generation and median cost, a log‑10 scale, allowing comparison of low‑ (e.g., phenobarbital) and high‑cost (e.g., cannabidiol) agents.

BRV: brivaracetam; CBZ: carbamazepine; CNB: cenobamate; ESL: eslicarbazepine, ESM: ethosuximide; EVER: everolimus; GBP: gabapentin; LEV: levetiracetam; LCM: lacosamide; LTG: lamotrigine; OXC: oxcarbazepine; PER: perampanel; PGB: pregabalin; PHT: phenytoin; PRM: primidone; RFM: rufinamide; STP: stiripentol; TPM: topiramate; VGB: vigabatrin; VPA: valproate acid; ZNS: zonisamide


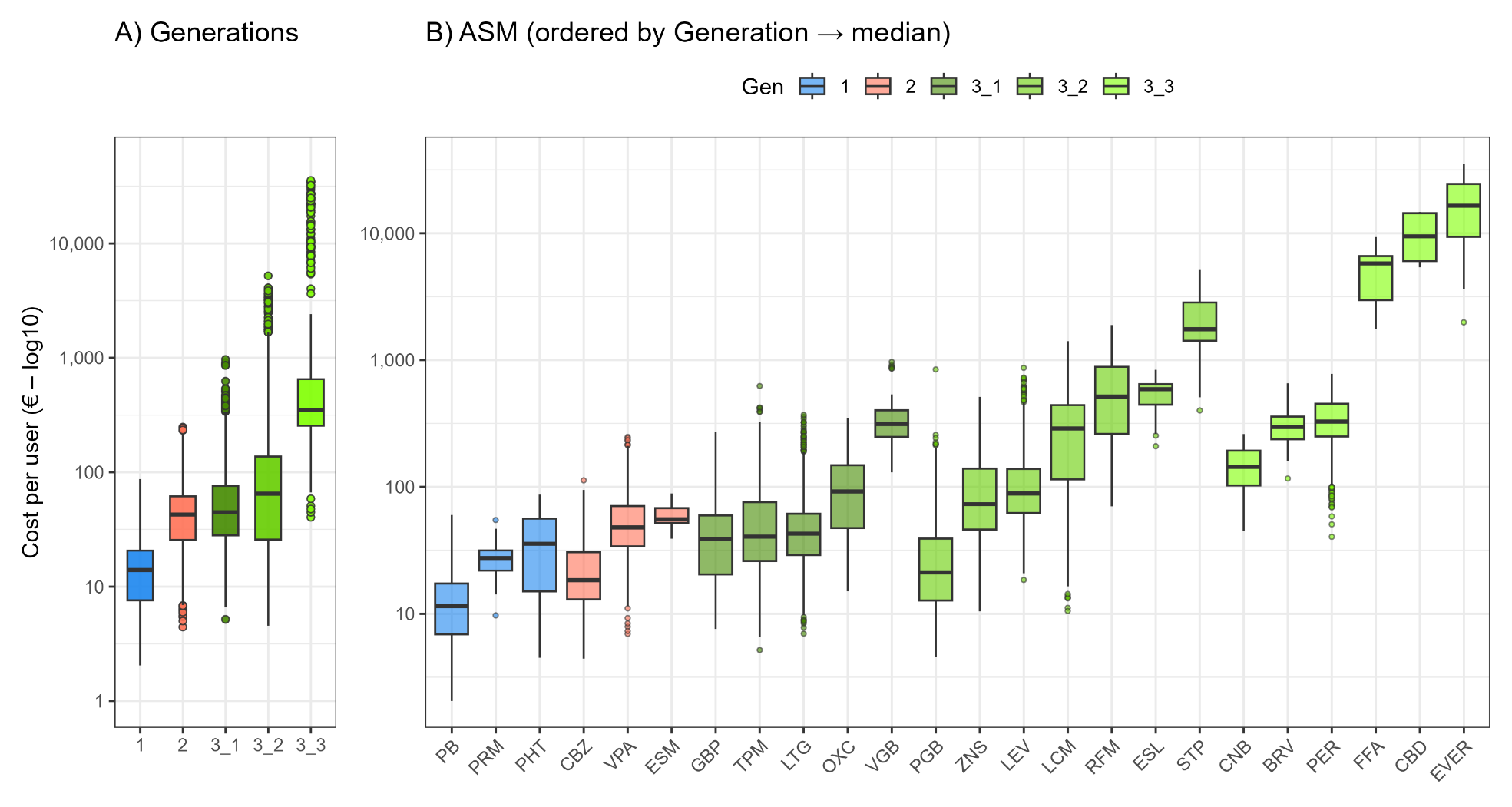


# Bibliography

1 O. M, R. B, M. LL-M, et al. Use of the French National Health Data System (SNDS) in pharmacoepidemiology: A systematic review in its maturation phase. Thérapies 2024.

2 Open Medic : base complète sur les dépenses de médicaments interrégimes. https://www.data.gouv.fr/datasets/open-medic-base-complete-sur-les-depenses-de-medicaments-interregimes/ (accessed Sept 18, 2025).

3 Zeileis A. Econometric Computing with HC and HAC Covariance Matrix Estimators. J Stat Softw 2004; 11: 1–17.

4 Hosmer DW, Lemeshow S, Sturdivant RX. Applied Logistic Regression: Third Edition. Applied Logistic Regression: Third Edition 2013; : 1–510.

5 McFadden D. Conditional logit analysis of qualitative choice behavior. 2021; published online Sept 27. https://www.academia.edu/53553324/Conditional_logit_analysis_of_qualitative_choice_behavior (accessed Sept 18, 2025).

6 Fox J, Monette G. Generalized collinearity diagnostics. J Am Stat Assoc 1992; 87: 178–83.

7 Simon N, Friedman J, Hastie T, Tibshirani R. Regularization Paths for Cox’s Proportional Hazards Model via Coordinate Descent. J Stat Softw 2011; 39: 1–13.

8 Colin CA, Pravin T. Regression Analysis of Count Data. Regression Analysis of Count Data, Second Edition 2013; : 1–567.

9 Stone M. Cross-Validatory Choice and Assessment of Statistical Predictions. J R Stat Soc Series B Stat Methodol 1974; 36: 111–33.

10 Van Der Weele TJ, Ding P. Sensitivity Analysis in Observational Research: Introducing the E-Value. Ann Intern Med 2017; 167: 268–74.

11 Jonckheere AR. A Distribution-Free k-Sample Test Against Ordered Alternatives. Biometrika 1954; 41: 133.

12 Naissances et taux de natalité | Insee. https://www.insee.fr/fr/statistiques/2381380 (accessed Sept 18, 2025).

13 Sullivan J, Benítez A, Roth J, et al. A systematic literature review on the global epidemiology of Dravet syndrome and Lennox–Gastaut syndrome: Prevalence, incidence, diagnosis, and mortality. Epilepsia 2024; 65: 1240–63.

14 Banque Nationale de Données Maladies Rares. Nombre de cas par maladie rare. 2024 https://www.bndmr.fr (accessed Sept 18, 2025).

15 SPINRAZA 12 mg sol inj - VIDAL. https://www.vidal.fr/medicaments/spinraza-12-mg-sol-inj-181979.html#precautions-particulieres-de-conservation (accessed Sept 18, 2025).

16 Stoke Announces Phase 3 EMPEROR Registrational Study for Zorevunersen in Dravet Syndrome | NeurologyLive - Clinical Neurology News and Neurology Expert Insights. https://www.neurologylive.com/view/stoke-announces-phase-3-emperor-registrational-study-zorevunersen-dravet-syndrome (accessed Sept 18, 2025).

17 ZOLGENSMA 2x10puissance13 génomes de vecteur/ml sol p perf - VIDAL. https://www.vidal.fr/medicaments/zolgensma-2x10puissance13-genomes-de-vecteur-ml-sol-p-perf-213453.html (accessed Sept 18, 2025).
